# A2 astrocyte polarization mediates ketogenic diet protection of blood–central nervous system barriers in experimental autoimmune encephalomyelitis

**DOI:** 10.4103/NRR.NRR-D-25-00062

**Published:** 2025-12-30

**Authors:** Qianye Zhang, Mingxiao Zheng, Hans-Christian Siebert, Qingpeng Wang, Ruiyan Zhang, Ning Zhang

**Affiliations:** 1 Shandong Key Laboratory of Applied Technology for Protein and Peptide Drugs, Institute of Biopharmaceutical Research, Liaocheng University, Liaocheng, Shandong Province, China; 2 RI-B-NT Research Institute of Bioinformatics and Nanotechnology, Kiel, Germany

**Keywords:** A1 astrocytes, A2 astrocytes, blood–brain barrier, blood–spinal cord barrier, experimental autoimmune encephalomyelitis, ketogenic diet, multiple sclerosis, nerve regeneration, neuroinflammation, NOD-, LRR- and pyrin domain-containing protein 3 (NLRP3) inflammasome

## Abstract

Disruption of the blood–brain barrier and blood-spinal cord barrier is a fundamental pathological feature of multiple sclerosis progression. The ketogenic diet has a high therapeutic potential for patients with multiple sclerosis. We previously reported that treating experimental autoimmune encephalomyelitis mice with ketogenic diet results in anti-neuroinflammation and neuroprotection. However, the impact of ketogenic diet administration on the blood–brain barrier/blood–spinal cord barrier in MS remains unclear. Here, we investigated the effects of ketogenic diet on the blood–brain barrier/blood–spinal cord barrier integrity and the possible underlying mechanisms. We established a 24-day continuous experimental autoimmune encephalomyelitis mouse model with or without ketogenic diet and performed β-hydroxybutyrate assay kit histological analysis, quantitative reverse transcription-polymerase chain reaction, and western blot to examine experimental autoimmune encephalomyelitis pathological hallmarks, glial cell activation status, and intracellular signaling pathway alterations. Our results showed that ketogenic diet inhibited demyelination, suppressed astrocyte and microglial activation, and modulated the balance of matrix metalloproteinases/tissue inhibitors of metalloproteinases in the central nervous system of experimental autoimmune encephalomyelitis mice. Ketogenic diet upregulated tight junction proteins (occludin, claudin-1, and ZO-1) and adherens junction proteins (VE-cadherin and β-catenin) in the spinal cord, cerebellum, and cortex of experimental autoimmune encephalomyelitis mice. Notably, we found that ketogenic diet protects the blood–brain barrier/blood–spinal cord barrier integrity by modulating astrocyte polarization from the A1 phenotype to A2 phenotype and modifying the inflammatory milieu (downregulating pro-inflammatory cytokines, including tumor necrosis factor-α, interleukin-1β, and interleukin-6, and upregulating anti-inflammatory cytokines such as transforming growth factor-β and interleukin-4) by inhibition of class I histone deacetylase 3/STAT3/nuclear factor kappa B (NF-κB)/NOD-, LRR- and pyrin domain-containing protein 3 and activation of PI3K/AKT signaling pathways. Furthermore, ketogenic diet downregulated key chemokines (C–X–C motif chemokine ligand 10, C–X–C motif chemokine ligand 12, C–C motif chemokine ligand 2, and C–C motif chemokine ligand 5) and receptor C–C motif chemokine receptor 2 expression throughout the central nervous system, suggesting an impaired capacity for leukocyte recruitment. Ketogenic diet suppressed astrocytic NOD-, LRR- and pyrin domain-containing protein 3 inflammasome activation, as evidenced by reduced NOD-, LRR- and pyrin domain-containing protein 3/glial fibrillary acidic protein co-localization. In summary, the ketogenic diet promotes neuroprotection in the experimental autoimmune encephalomyelitis model by inhibiting A1 astrogliogenesis and protecting the integrity of the blood–brain barrier/blood-spinal cord barrier.

## Graphical abstract

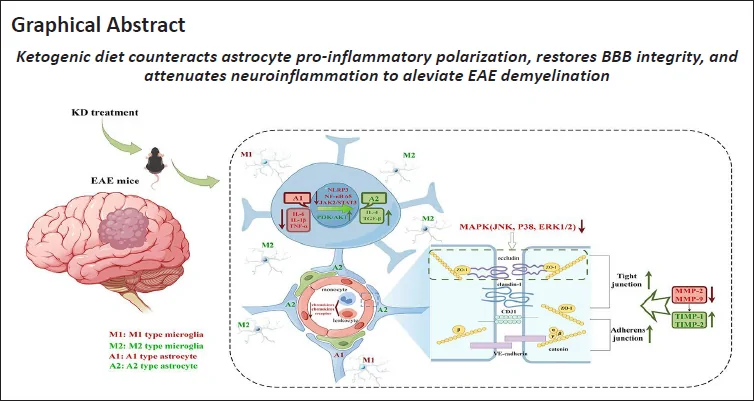

## Introduction

Multiple sclerosis (MS) is an inflammatory neurological disease of the central nervous system (CNS) that is characterized by demyelination, glial activation, and axonal loss (Arya et al., 2024). Several immunomodulators are available for relapsing-remitting MS but are associated with poor adherence; moreover, no therapies effectively halt disease progression (Charabati et al., 2023). Therefore, C-X-C motif chemokine ligand 12 (CXCL12) developing safe and effective treatments to alleviate MS is critical.

In MS, a central event in disease pathogenesis is the breakdown of the blood–brain barrier (BBB) or/and blood-spinal cord barrier (BSCB), which permits the infiltration of peripheral immune cells into the CNS (Bhaskaran et al., 2023; Luo et al., 2023). This breach initiates a cascade of neuroinflammation, leading to widespread demyelination, the formation of characteristic sclerotic plaques, and subsequent axonal injury (Li et al., 2022a; Chung et al., 2023). The inflammatory milieu further disrupts CNS homeostasis by impairing oligodendrocyte function, thereby hindering remyelination and repair, which directly correlates with irreversible neurological deficits (Fang et al., 2022). Central to the barrier disruption is the degradation of tight junction (TJ) and adherent junction (AJ) proteins, which are essential structural components of the BBB/BSCB. Consequently, therapeutic strategies aimed at preserving BBB/BSCB integrit, such as targeting specific adhesion molecules, inhibiting matrix metalloproteinases (MMPs), or directly reinforcing TJ and AJ complexes, represent promising approaches to limit immune cell trafficking, mitigate neuroinflammation, and ultimately attenuate CNS damage in MS (Lutz et al., 2017; Jin et al., 2021).

Astrocytes and microglia also contribute to the maintenance of BBB/BSCB, with astrocytic end-feet extensively enveloping and communicating with the endothelial cells (Fæste et al., 2022; Huang et al., 2022; Takahashi, 2022). However, during MS progression, both microglia and astrocytes become activated.and release pro-inflammatory factors such as interleukin (IL)-6, IL-1β, and tumor necrosis factor-α (TNF-α), which increases BBB/BSCB permeability and facilitates the invasion of peripheral immune cells like pathogenic Th1 and Th17 lymphocytes (Kempuraj et al., 2016; Li et al., 2021a; Kunkl et al., 2022; Zhao et al., 2024). This neuroinflammatory cascade is further amplified by cellular cross-talk, wherein M1-polarized microglia secrete mediators including C1q, IL-1β, and TNF-α that drive astrocytes into a reactive state. Reactive astrocytes undergo astrogliosis, characterized by disrupted end-feet connections, excessive proliferation, and glial scar formation. They are broadly polarized into a neurotoxic A1 phenotype, which releases pro-inflammatory factors and MMPs that damage neurons and oligodendrocytes, and a neuroprotective A2 phenotype (Chang et al., 2023; Gotoh et al., 2023). Notably, inhibition of the NLRP3 inflammasome mediates protection against chronic intermittent hypoxia-induced brain injury by promoting a shift in astrocyte phenotype from A1 to A2 (She et al., 2022). Therefore, therapeutic strategies that restrict microglial and astrocyte activation, promote the protective M2/A2 phenotypes, and inhibit the detrimental M1/A1 phenotypes, may restore BBB/BSCB integrity and slow MS disease progression.

MMPs are a family of enzymes responsible for degrading various extracellular matrix components, including myelin proteins such as myelin basic protein (MBP), and their activity is naturally regulated by tissue inhibitors of metalloproteinases (TIMPs), which help maintain proteolytic balance. Crucially, the dynamic between MMPs and TIMPs is essential for the integrity of the BBB. However, in MS and its experimental autoimmune encephalomyelitis (EAE) model, MMPs such as MMP-9 and MMP-2 are significantly upregulated (Ghasemi Sakha et al., 2020; Fehlhofer et al., 2024), which leads to BBB disruption and active demyelination. In contrast, studies in the EAE model demonstrate that TIMPs exert protective effects by inhibiting MMP activity and suppressing demyelination, thereby preventing broader disease-related tissue damage (Hansmann et al., 2012).

The ketogenic diet (KD), characterized by hepatic ketogenesis and elevated systemic levels of its major metabolite β-hydroxybutyrate (β-HB), exerts multiple beneficial effects on the CNS (Li et al., 2021b; Zhu et al., 2022). Beyond serving as an alternative energy substrate for the brain during glucose deprivation, β-HB improves memory and synaptic plasticity through epigenetic mechanisms, primarily via inhibition of class I histone deacetylases (HDAC1/3/4) (Xie et al., 2015). Accumulating evidence indicates that the KD and β-HB help preserve BBB integrity by suppressing neuroinflammation, attenuating pathological glial activation, restoring metabolic balance, and stabilizing BBB components (Wang et al., 2021, 2023; Sun et al., 2023). Experimental support for its efficacy comes from the EAE mouse model (a widely used preclinical model of MS), where KD intervention was shown to attenuate demyelination and clinical severity, potentially through immunomodulatory effects such as expanding regulatory T cells and suppressing pro-inflammatory T cell subsets (Choi et al., 2016). Emerging clinical evidence further suggests that the KD may offer therapeutic benefits for patients with relapsing-remitting MS, with a recent study confirming its safety and preliminary efficacy (Brockhoff et al., 2023). However, the specific effect of KD on BBB/BSCB integrity in MS, particularly its role in modulating astrocyte polarization and the associated molecular mechanisms, remains unclear. Thus, we explored the effects of KD on astrocyte phenotypes and BBB/BSCB integrity in EAE mice and investigated the possible molecular mechanism of KD administration.

## Methods

### Animals

Female C57BL/6 mice (6 weeks old, 20–23 g) were obtained from Jinan Pengyue Experimental Animal Breeding Center (Laboratory Animal Production License Number: SCXK (Lu) 20220006) and acclimatized under controlled conditions (23 ± 2°C, 50% ± 10% humidity, 12-hour light/dark cycle) with free access to food and water (Alvarez-Sanchez and Dunn, 2023; Guadalupi et al., 2024). Mice were pair-housed after random assignment. Prior to experimental procedures, animals received intraperitoneal anesthesia (ketamine/xylazine, 100/10 mg/kg body weight). All experimental protocols were approved by Liaocheng University’s Institutional Animal Care and Use Committee (approval No. LL-2022111030) and conformed to the NIH Guide for the Care and Use of Laboratory Animals.

### Induction of experimental autoimmune encephalomyelitis and ketogenic diet administration

The mice were randomized into three groups (*n* = 8/group): the control group (standard AIN-93 diet), the EAE group (EAE model, standard diet), and the EAE + KD group (EAE model, administered KD). The standard diet (AIN-93) was provided by Beijing HFK Bioscience Co., Ltd. (Beijing, China). The KD composition (**Additional Table 1**) followed established protocols (Liu C et al., 2020).

**Additional Table 1:** Composition of the SD and KD used in this study

	SD		KD	

	Weight (g/kg)	Energy density (kcal/g)	Weight (g/kg)	Energy density (kcal/g)
Casein	200.00	0.800	194.00	0.776
Sucrose	132.00	0.528	/	/
Maltodextrin	100.000	0.400	/	/
Corn starch	397.500	1.590	/	/
L-Cystine	3.000	0.012	3.000	0.012
Cellulose	50.000	/	23.500	/
V1002 vitamin mix^a^	10.000	0.040	10.000	0.040
M1003G mineral mix	35.000	/	35.000	/
Choline bitartrate	2.500	/	2.500	/
Tert-butyl-hydroquinone	0.014	/	0.014	/
Soybean oil	70.000	0.630	85.000	0.765
Medium-chain triglycerides oil	/	/	370.000	3.330
Medium-chain triglycerides powder^b^	/	/	277.000	1.830
Total (g)	1000.014	4.000	1000.014	6.753
Ketogenic ratio	0.080:1		3.060:1	

The standard diet (SD) was based on the composition of AIN-93G. ^a^Containing 100% (w/w) sucrose. ^b^ontaining 72% (w/w) fat, 2.2% (w/w) protein, 1.2% (w/w) sucrose, and 22.2% (w/w) cellulose. KD: Ketogenic diet; SD: standard AIN-93 diet.

MOG35-55 peptide (2 mg/mL in PBS, GL Biochem, Shanghai, China) was emulsified with complete Freund’s adjuvant (CFA, DIFCO Laboratories, UK) at a 1:1 ratio. To induce EAE, mice received subcutaneous injections (100 µL) at the dorsal neck and tail base. Pertussis toxin (200 ng, Calbiochem, Darmstadt, Germany) was administered intraperitoneally immediately post-immunization and again after 48 hours. To examine the effects of a KD on EAE, a 24-day continuous EAE model was employed. The KD feeding and EAE induction were initiated concurrently; specifically, mice began receiving the KD on the same day as immunization. At the endpoint of the 24-day experiment, all mice were euthanized. Euthanasia was performed in accordance with the the AVMA Guidelines for the Euthanasia of Animals. Briefly, mice were deeply anesthetized with an intraperitoneal injection of sodium pentobarbital (150 mg/kg) and then transcardially perfused with fixative or saline, depending on the subsequent tissue processing needs. Tissue collection was performed as follows: a subset of mice from each group was transcardially perfused with 4% paraformaldehyde. The brain and spinal cord tissues from these animals were post-fixed in paraformaldehyde and subsequently prepared for histochemical analysis. For the remaining mice, the brain was microdissected into specific regions—such as the cerebellum and cortex, and the spinal cord was carefully excised. These tissues were immediately flash-frozen in liquid nitrogen and stored at –80°C for subsequent biochemical analyses.

EAE clinical symptoms were evaluated daily using a validated scoring scale: 0 (asymptomatic), 1 (tail paralysis), 2 (impaired coordination), 3 (partial hindlimb paralysis), 4 (complete paralysis), or 5 (moribund state) (Lindsay et al., 2022).

### Measurement of β-hydroxybutyrate

The level of β-HB was assessed using a mouse β-HB assay kit (JM-11641M2, JINGMEI, Shenzhen, China). Briefly, standard substances, mouse samples (spinal cord, cerebellum, and cortex), and horseradish peroxidase (HRP)-labeled detection antibodies (JM-11641M2) were successively added to the pre-coated β-HB antibody-coated micropores, followed by incubation at 37°C for 1 hour. After five washes, tetramethyl benzidine (JM-11641M2) was added for color development. Tetramethyl benzidine undergoes enzymatic conversion by peroxidase, initially producing a blue chromogen that shifts to yellow following acid stop solution addition. Optical density measurements at 450 nm were acquired with a Spark microplate analyzer (Tecan Group Ltd., Männedorf, Switzerland), enabling sample concentration calculations.

### Immunofluorescence

Tissue sections (spinal cord, cerebellum, and cortex) were processed for immunofluorescence as previously described (Zhang et al., 2025). Following deparaffinization and antigen retrieval (citrate buffer, pH 6), samples were blocked with 5% BSA in PBS-T (0.3% Triton X-100) for 1 hour at 37°C. Samples were incubated with the following primary antibodies overnight at 4°C: glial fibrillary acidic protein (GFAP; mouse, 1:500, Cat# 60190-1-Ig, Proteintech, Wuhan, China) and ionized calcium-binding adapter molecule 1 (Iba-1; mouse, 1:500, Cat# ab283319, Abcam, Cambridge, UK). CD31 (mouse, 1:500, Cat# ab9498, Abcam). Sections were washed with PBS three times and incubated with Coralite 594–conjugated goat anti-mouse antibody (1:400, Cat# SA00013-3, Proteintech) at room temperature (22-25°C) for 1 hour. Nuclei were counterstained with 4′,6′-diamidino-2-phenylin dole (DAPI; 0.5 µg/mL, Cat# C0065, Solarbio, Beijing, China) for 5 minutes. Fluorescent signals were captured using an Olympus BX53 epifluorescence microscope (Tokyo, Japan). Quantitative analysis of fluorescence intensity was performed by calculating mean gray values using ImageJ software (v1.8.0, National Institutes of Health, USA).

### Immunofluorescence double-labeling

Double-label immunofluorescence was performed following published methods (Zhang et al., 2024). Sections of paraffin-embedded tissues (spinal cord, cerebellum, and cortex) were dewaxed, and antigen retrieval was performed in citrate buffer (pH 6). Sections were co-incubated with antibodies against Amigo2 (rabbit, 1:300, Cat# bs-11450R, Bioss, Beijing, China), S100a10 (rabbit, Cat# 11250-1-AP, 1:300, Proteintech), NOD-, LRR- and pyrin domain-containing protein 3 (NLRP3; rabbit, 1:300, Cat# A5652, ABclonal, Wuhan, China), CD16/32 (rabbit, 1:500, Cat# ab223200, Abcam), Arg1 (rabbit, 1:300, Cat# 16001-1-AP, Proteintech), phosphoinositide 3-kinase (PI3K; mouse, 1:300, Cat# 67071-1-Ig, Proteintech), protein kinase B (AKT; mouse, Cat# 60203-2-Ig, 1:200, Proteintech) and antibodies against GFAP (rabbit, 1:500, Cat# 16825-1-AP, Proteintech; mouse, 1:500, Cat# 60190-1-Ig, Proteintech) or Iba-1 (mouse, 1:500, Cat# ab283319, Abcam) overnight at 4°C. Following PBS washes, sections were incubated for 1 hour at room temperature (protected from light) with fluorescent secondary antibodies: Coralite 488–conjugated goat anti-rabbit IgG (1:400, Cat# SA00013-2, Proteintech) and Coralite 594–conjugated goat anti-mouse IgG (1:400, Cat# SA00013-3, Proteintech). Nuclear counterstaining was performed using DAPI (0.5 µg/mL, 15 minutes). After PBS washes, slides were mounted with Dako fluorescence mounting medium (S3023) and coverslipped. Fluorescence imaging was conducted using an Olympus IX73 inverted microscope; quantitative analysis of signal intensity was performed using ImageJ 1.8.0.

### Immunohistochemistry

Paraffin sections of tissues (spinal cord, cerebellum, and cortex) were dewaxed, and antigen retrieval was conducted in citrate buffer (pH 6.0), followed by blocking with BSA. Sections were incubated with the following primary antibodies overnight at 4°C: Claudin5 (1:500, rabbit, Cat# 29767-1-AP, Proteintech) and occludin (rabbit, 1:2000, Cat# 27260-1-AP, Proteintech). Sections were then incubated with biotin-conjugated secondary antibody (Cat# RGAR011, Proteintech) for 25 minutes at room temperature. DAB coloration was performed for 25 minutes, followed by counterstaining with hematoxylin (Cat# H8070, Solarbio) for 1 minute. Images were taken using an epifluorescence inverted microscope (Olympus IX73, Japan). Images were analyzed using ImageJ software (version 1.8.0, NIH, Bethesda, MD, USA).

### Western blotting

Protein extracts from spinal cord, cerebellum, and cortex were prepared using RIPA buffer (Beyotime, Shanghai, China) containing 1× protease inhibitor and 1× phosphatase inhibitor. Protein quantification was performed using the BCA assay (Beyotime). Samples (25 µg) were resolved on 8%–12% SDS-PAGE gels and electrotransferred to PVDF membranes (Shenggong, Shanghai, China). After blocking with 5% non-fat milk for 2 hours at room temperature, the membranes were washed with TBST (Tris-buffered saline with 0.2% Tween 20, pH 7.4) and then probed with primary antibodies (**Additional Table 2**) overnight at 4°C. Membranes were then incubated with HRP-conjugated anti-rabbit IgG (1:10,000, SA00001-2, Proteintech) for 1.5 hours at room temperature. Chemiluminescent signals were captured using a Tanon detection system (Tanon, Shanghai, China) and quantified relative to β-actin using ImageJ software.

**Additional Table 2:** Antibodies used for western blot analysis

Protein	Host	Molecular weight (kDa)	Manufacturer	Dilution	Cat#
Oligo2	Rabbit	32	Abcam	1:1000	ab-220796
NG2	Rabbit	251	Proteintech	1:2000	55027-1-AP
MBP	Rabbit	40	Proteintech	1:1000	15089-1-AP
CNPase	Rabbit	48	Proteintech	1:1000	13427-1-AP
ZO-1	Rabbit	230	Proteintech	1:8000	21773-1-AP
Claudin-1	Rabbit	22	Abcam	1:2000	ab211737
Occludin	Rabbit	59	Proteintech	1:8000	27260-1-AP
VE-cadherin	Rabbit	130	Bioss	1:1000	Bs-0878R
β-catenin	Rabbit	92	Proteintech	1:8000	17565-1-AP
JNK	Rabbit	48	Proteintech	1:5000	17572-1-AP
p-JNK	Rabbit	48	Proteintech	1:5000	80024-1-RR
ERK1/2	Rabbit	42	Proteintech	1:10000	ab184699
p-ERKl/2	Rabbit	42	Wanleibio	1:750	WLP1512
P38	Rabbit	38	Wanleibio	1:500	WL00764
p-P38	Rabbit	38	Wanleibio	1:750	WLP1576
GFAP	Rabbit	50	Proteintech	1:5000	16825-1-AP
Iba-1	Rabbit	17	Proteintech	1:1000	10904-1-AP
Amigo2	Rabbit	54	Bioss	1:1000	bs-11450R
IL-6	Rabbit	20	Proteintech	1:1000	21865-1-AP
TNF-α	Rabbit	26	Proteintech	1:1000	17590-1-AP
PTX3	Rabbit	42	Proteintech	1:1000	13797-1-AP
IL-4	Rabbit	14	Bioss	1:1000	bs-0581R
CD86	Rabbit	80	Boster	1:5000	A00220-4
CD16+CD32	Rabbit	40	Abcam	1:1000	ab-223200
Arg1	Rabbit	36	Proteintech	1:8000	16001-1-AP
TGFβ1	Rabbit	44	Proteintech	1:1000	21898-1-AP
CD206	Rabbit	166	Proteintech	1:1000	18704-1-AP
HDAC3	Rabbit	48	Proteintech	1:2000	10255-1-AP
NLRP3	Rabbit	115	Abcam	1:1000	ab263899
cleaved caspaesel	Rabbit	25	Abcam	1:2000	ab207802
IL-18	Rabbit	22	Proteintech	1:2000	10663-1-AP
JAK2	Rabbit	131	Abcam	1:5000	ab108596
p-JAK2	Rabbit	130	Abcam	1:5000	ab32101
STAT3	Rabbit	88	Abcam	1:2000	ab109085
p-STAT3	Rabbit	88	Abcam	1:5000	ab76315
MyD88	Rabbit	33	Proteintech	1:2000	23230-1-AP
IKBα	Rabbit	38	Abcam	1:3000	ab32518
p-IKBα	Rabbit	36	Abcam	1:3000	ab133462
NF-κB	Rabbit	65	Proteintech	1:4000	10745-1-AP
P-NF-κB	Rabbit	75	Abcam	1:2000	ab76302
cleaved-IL-1β	Rabbit	23	Abcam	1:1000	ab216995
PI3K	Rabbit	123	Proteintech	1:500	20584-1-AP
p-PI3K	Rabbit	113	Bioss	1:1000	bs-6417R
AKT	Rabbit	56	Abcam	1:1000	ab38449
p-AKT	Rabbit	58	Proteintech	1:5000	80455-1-AP
MMP-2	Rabbit	72	Proteintech	1:1000	10373-2-AP
MMP-9	Rabbit	78	Proteintech	1:1000	10375-2-AP
TIMP-1	Rabbit	23	Abcam	1:1000	ab179580
TIMP-2	Rabbit	24	Abcam	1:1000	ab180630
β-actin	Rabbit	42	Proteintech	1:3000	81115-1-RR

GFAP: Glial fibrillary acidic protein; HDAC: class I histone deacetylase; Iba-1: ionized calcium-binding adapter molecule 1; IKBα: inhibitor of NF-κBα; IL: interleukin; MBP: myelin basic protein; MMP: matrix metalloproteinase; NF-κB: nuclear factor kappa B; NLRP3: NOD-, LRR- and pyrin domain-containing protein 3; TGF-β: transforming growth factor-β; TIMP: tissue inhibitors of metalloproteinase; TNF-α: tumor necrosis factor-α.

### Quantitative reverse transcription-polymerase chain reaction

Total RNA was isolated from CNS tissues using the Trans RNeasy Kit (TransGen Biotech, Beijing, China), as previously described (Sun et al., 2023). cDNA synthesis was performed using Roche Transcriptor reagents (Servicebio, Wuhan, China). Gene expression analysis was conducted on a StepOne^TM^ Real-Time PCR System with SYBR Green Chemistry (Servicebio); primer sequences are listed in **Additional Table 3**. Amplification specificity was confirmed by melt curve analysis, and relative quantification was determined by the 2^–ΔΔCt^ method using glyceraldehyde-3-phosphate dehydrogenase (GAPDH) mRNA as reference.

**Additional Table 3:** Primer sequences for quantitative reverse transcription-polymerase chain reaction

Primer name	Sequence (5’-3’)
Olig1	F: CTAAGAGCTGCTCCCCAACAGT R: CCTGGGCTTTCTAGACCTCCT
Olig2	F: TCCACCAAGAAAGACAAGAAGCAGA R: ATGGCGATGTTGAGGTCGTGC
Occludin	F: GTCCGTGAGGCCTTTTGA R: GGTGCATAATGATTGGGTTTG
ZO-1	F: CGCGGAGAGAGACAAGATGT R: GAAGCGTCACTGTGTGCTGT
VE-cadherin	F: GTGGATGAGCCCCCTGTCT R: CAGCGGTTTCTTCTCGTTTTCT
C3	F: AGCAGGTCATCAAGTCAGGC R: GATGTAGCTGGTGTTGGGCT
GBP2	F: GATTGGCCCGCTCCTAAGAA R: TTGACGTAGGTCAGCACCAG
SPHK1	F: GGCTCTGCAGCTCTTCCAGAG R: CTCCTCTGCACACACCAGCTC
PTX3	F: CGCTGTGCTGGAGGAACT R: ATTGCTGTTTCACAACCTG
S100a10	F: TGAGAGTGCTCATGGAACGG R: AGAAAGCTCTGGAAGCCCAC
CXCL10	F: GCTGCCGTCATTTTCTGC R: TCTCACTGGCCCGTCATC
CXCL2	F: AAGTTTGCCTTGACCCTGAA R: AGGCACATCAGGTACGATCC
CCL2	F: TTGTCACCAAGCTCAAGAGAGA R: GAGGTGGTTGTGGAAAAGGTAG
CCL5	F: TGCTGCTTTGCCTACCTC R: CTTGAACCCACTTCTTCTCT
GAPDH	F: GAGGCCGGTGCTGAGTATGT R: GGTGGCAGTGATGGCATGGA
CXCL12	F: GCGCTCTGCATCAGTGAC R: TTTCAGATGCTTGACGTTGG
CCR2	F: TGACAGGCACAGATGAATGG R: ATCATCTCCTGGCTGAATGC

F: Forward; GAPDH: glyceraldehyde-3-phosphate dehydrogenase; R: reverse.

### Statistical analysis

Statistical analysis was performed using SPSS software (v25.0, IBM, Armonk, NY, USA). Parametric assumptions were verified through Kolmogorov–Smirnov normality testing (*P* > 0.05) and Levene’s variance homogeneity assessment. For parametric comparisons, one-way analysis of variance with Tukey’s *post hoc* test was applied. Non-parametric analyses used Kruskal–Wallis testing followed by Dunn’s procedure with Bonferroni adjustment. Data requiring normalization were appropriately transformed. Data are reported as mean ± standard error of the mean. *P* values < 0.05 indicated statistical significance.

## Results

### Ketogenic diet administration reduces neurological scores and increased β-hydroxybutyrate levels in experimental autoimmune encephalomyelitis mice

**Figure 1:**
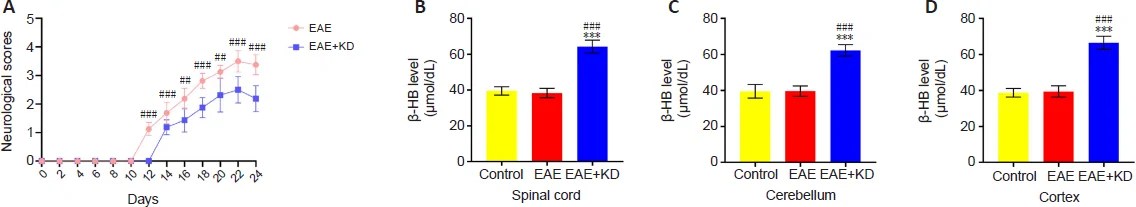
Neurological scores and effect of KD on the levels of β-HB in the spinal cord, cerebellum and cortex of EAE mice. (A) Comparison of neurological scores between EAE and EAE + KD. (B–D) Quantitative analysis of β-HB levels in the spinal cord (B), cerebellum (C), and cortex (D). Data are presented as the mean ± SEM (*n* = 4). ***P < 0.001, *vs.* control group; ###*P* < 0.001, *vs.* EAE + KD group (one-way analysis of variance with Tukey’s *post hoc* test). EAE: Experimental autoimmune encephalomyelitis; KD: ketogenic diet; β-HB: β-hydroxybutyrate.

As assessed by neurological scoring from day 12 to 24 post-induction, EAE-induced mice exhibited significantly higher deficit scores than controls, while KD administration significantly ameliorated these deficits (day 12, *P* < 0.001; day 14, *P* < 0.001; day 16, *P* < 0.01; day 18, *P* < 0.001; day 20, *P* < 0.01; day 22, *P* < 0.001; day 24, *P* < 0.001; **Figure 1A**), demonstrating that KD administration reduces disease severity in the model. To evaluate the neuroprotective potential of a KD in the EAE model, mice were allocated to KD or control diet groups for 24 days post-immunization. We measured β-HB concentrations (**Additional Table 4**) to verify successful dietary-induced ketosis, thereby establishing the essential metabolic context for subsequent analysis of the diet’s protective effects. When comparing the EAE model group to the healthy control group, no significant differences in β-HB levels were observed in the spinal cord, cerebellum, or cortex. However, the levels of β-HB are elevated in the spinal cord (*P* < 0.001), cerebellum (*P* < 0.001), and cortex (*P* < 0.001) of EAE + KD mice compared with EAE mice (**Figure 1B–D**). Furthermore, our previous findings showed that there were no differences in the body weights of mice among the control, EAE, and EAE + KD groups, indicating that KD administration did not affect final body weight (Sun et al., 2023).

**Additional Table 4:** Levels of β-HB in the spinal cord, cerebellum, and cortex

β-HB concentration (μmol/dL)	Spinal cord			Cerebellum			Cortex		
		
	Control	EAE	EAE+KD	Control	EAE	EAE+KD	Control	EAE	EAE+KD
Sample 1	40.1475	40.8654	64.6235	35.1422	36.8616	58.623	38.4272	36.6485	67.0441
Sample 2	36.9499	38.512	67.71	41.9358	39.506	63.716	41.2431	42.8478	69.9319
Sample 3	41.4143	35.5843	60.5143	41.4144	42.5763	64.5133	36.403	38.8085	62.616
Sample 4	40.2398	38.2746	63.2847	40.2123	40.1647	62.4918	37.8364	39.2837	65.2334
Sample 5	40.2483	39.2934	67.2847	41.4835	40.5827	66.3582	38.5848	39.5828	65.2948
Sample 6	39.5938	40.5982	66.6594	39.2928	40.5992	65.6764	38.8955	40.6636	65.3827
Sample 7	38.7937	40.6356	67.8646	38.9647	41.5436	66.4225	38.8754	39.85866	67.5367
Sample 8	39.6646	38.5437	63.5643	41.4968	40.4562	68.5872	39.5976	41.4975	65.6478

EAE: Experimental autoimmune encephalomyelitis; KD: ketogenic diet; β-HB: β-hydroxybutyrate.

### Ketogenic diet administration reduces demyelination in experimental autoimmune encephalomyelitis mice

**Figure 2:**
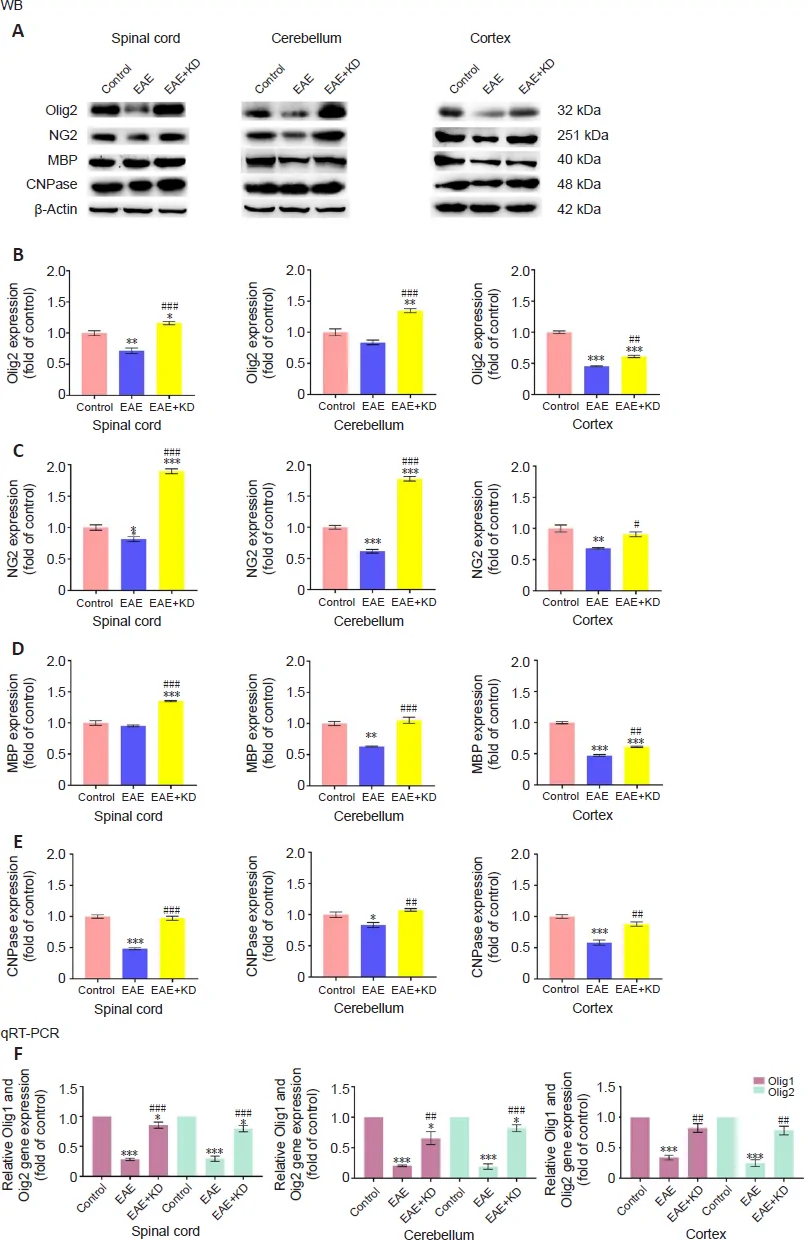
Effect of KD on demyelination in the spinal cord, cerebellum and cortex of EAE mice. (A) Representative western blot images of Olig2, NG2, MBP, and CNPase and β-actin in different groups. β-actin was used as the internal control for normalization. Quantitative western blot results for protein expression of oligodendrocyte lineage markers, including Olig2 (B), NG2 (C), MBP (D), and CNPase (E), in the spinal cord, cerebellum and cortex, respectively. (F) qRT-PCR analysis of Olig1 and Olig2 mRNA transcripts in the spinal cord, cerebellum and cortex in the different groups. Data are presented as the mean ± SEM (*n* = 4). *P < 0.05, **P < 0.01, ***P < 0.001, *vs*. control group; #*P* < 0.05, ##*P* < 0.01, ###*P* < 0.001, *vs*. EAE group (one-way analysis of variance with Tukey’s *post hoc* test). EAE: Experimental autoimmune encephalomyelitis; KD: ketogenic diet; MBP: myelin basic protein; qRT-PCR: quantitative reverse transcription-polymerase chain reaction.

EAE mice exhibit infiltration of inflammatory cells in the spinal cord and significant demyelination (Sun et al., 2023). To explore the neuroprotective effects of the KD on EAE mice, we examined key components of the oligodendrocyte lineage and myelin integrity in the spinal cord, cerebellum, and cortex via western blot analysis. We utilized four specific markers: Olig2 (expressed in both oligodendrocyte precursor cells and mature oligodendrocytes), NG2 (labeling oligodendrocyte progenitor cells), and the mature myelin proteins MBP and CNPase, which are essential structural components of the myelin sheath and thus serve as direct molecular indicators of myelination levels. Our results showed that compared with the control group, the EAE model group exhibited a significant downregulation in the protein levels of Olig2 (spinal cord, *P* < 0.01; cortex, *P* < 0.001) and NG2 (spinal cord, *P* < 0.05; cerebellum, *P* < 0.001; cortex, *P* < 0.01), alongside a marked decrease in the expression of the myelination-associated markers MBP (cerebellum, *P* < 0.01; cortex, *P* < 0.001) and CNPase (spinal cord, *P* < 0.001; cerebellum, *P* < 0.05; cortex, *P* < 0.001). However, administration of the KD to EAE mice notably reversed these alterations, leading to increased expression of Olig2 (spinal cord, *P* < 0.001; cerebellum, *P* < 0.001; cortex, *P* < 0.01) and NG2 (spinal cord, *P* < 0.001; cerebellum, *P* < 0.001; cortex, *P* < 0.05), and significantly elevating the levels of MBP (spinal cord, *P* < 0.001; cerebellum, *P* < 0.001; cortex, *P* < 0.01) and CNPase (spinal cord, *P* < 0.001; cerebellum, *P* < 0.01; cortex, *P* < 0.01) compared with the untreated EAE group (**Figure 2A–E**).

The Olig1 and Olig2 transcription factors are required for oligodendrocyte lineage development *in vivo* (Verma et al., 2023). We next examined whether the KD affected the mRNA levels of Olig1 and Olig2. Quantitative reverse transcription-polymerase chain reaction (qRT-PCR) analysis revealed that the mRNA expression levels of both Olig1 and Olig2 were significantly downregulated in the EAE group compared with the control group (Olig1 [spinal cord, *P* < 0.001; cerebellum, P < 0.001; cortex, *P* < 0.001]; Olig2 [spinal cord, *P* < 0.001; cerebellum, *P* < 0.001; cortex, *P* < 0.001]). This reduction was effectively reversed by KD administration, as evidenced by a significant upregulation of both transcripts in the EAE + KD group compared with the EAE group (Olig1 [spinal cord, *P* < 0.001; cerebellum, *P* < 0.01; cortex, *P* < 0.01]; Olig2 [spinal cord, *P* < 0.001; cerebellum, *P* < 0.001; cortex, *P* < 0.01]; **Figure 2F**). Collectively, these findings indicate that the KD attenuates demyelination while enhancing oligodendrocyte precursor cell expansion, thereby preserving white matter integrity in the EAE model.

### The ketogenic diet supports blood–brain barrier and blood-spinal cord barrier integrity

**Figure 3:**
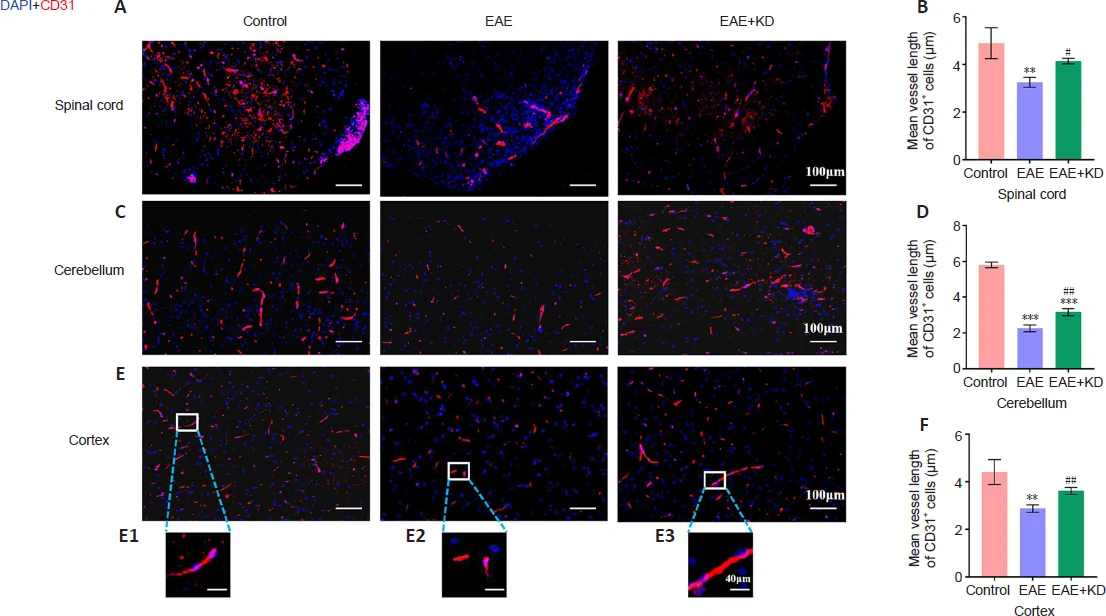
CD31 immunofluorescence staining of the spinal cord, cerebellum and cortex of EAE mice. Immunofluorescence staining of CD31 in the spinal cord (A), cerebellum (C), and cortex (E). Scale bars: 100 μm in A, C, and E; 40 μm in E1–E3 (magnified images of the white boxes from E). Quantification of CD31^+^ cells in the spinal cord (B), cerebellum (D), and cortex (F) of the three groups is presented as the mean vessel length. Data are presented as the mean ± SEM (*n* = 4). **P < 0.01, ***P < 0.001, *vs*. control group; #*P* < 0.05, ##*P* < 0.01, *vs.* EAE group (one-way analysis of variance with Tukey’s *post hoc* test). EAE: Experimental autoimmune encephalomyelitis; KD: ketogenic diet.

We next investigated the influence of the KD on BBB and BSCB impairment in EAE mice. CD31 (PECAM-1), a biomarker of endothelial cells (Raina et al., 2022), contributes to the regulation of BBB and BSCB integrity (Wimmer et al., 2019). Immunofluorescence staining showed that control mice had continuous CD31 staining in the spinal cord, cerebellum, and cortex, whereas EAE mice showed gaps or even absence of CD31 reactivity (**Figure 3A**, **C**, and **E**). In EAE + KD mice, CD31 staining appeared more continuous. Furthermore, the mean vessel length of CD31^+^ cells was significantly increased in the EAE + KD group compared with the EAE group, confirming enhanced vascular integrity (spinal cord, *P* < 0.05, cerebellum, *P* < 0.01, and cortex, *P* < 0.01; **Figure 3B**, **D**, and **F**, respectively).

TJ and AJ are important factors responsible for BBB and BSCB integrity (Vignone et al., 2022). TJ is composed of several proteins, including ZO-1, occludin, Claudin-1, and Claudin-5; AJ is mainly composed of VE-cadherin, β-catenin, and E-cadherin (Naser et al., 2023; Villalba et al., 2023). Immunohistochemical analysis revealed that the EAE group exhibited a significant downregulation of the TJ proteins Claudin-5 and occludin across the spinal cord (*P* < 0.001), cerebellum (*P* < 0.001), and cortex (*P* < 0.001) compared with the control group. However, KD administration significantly increased Claudin-5 (spinal cord, *P* < 0.001; cerebellum, *P* < 0.001; cortex, *P* < 0.01) and occludin (spinal cord, *P* < 0.05; cerebellum, *P* < 0.001; cortex, *P* < 0.01) expression in the EAE + KD group compared with the EAE group (**Additional Figure 1**).

**Figure 4:**
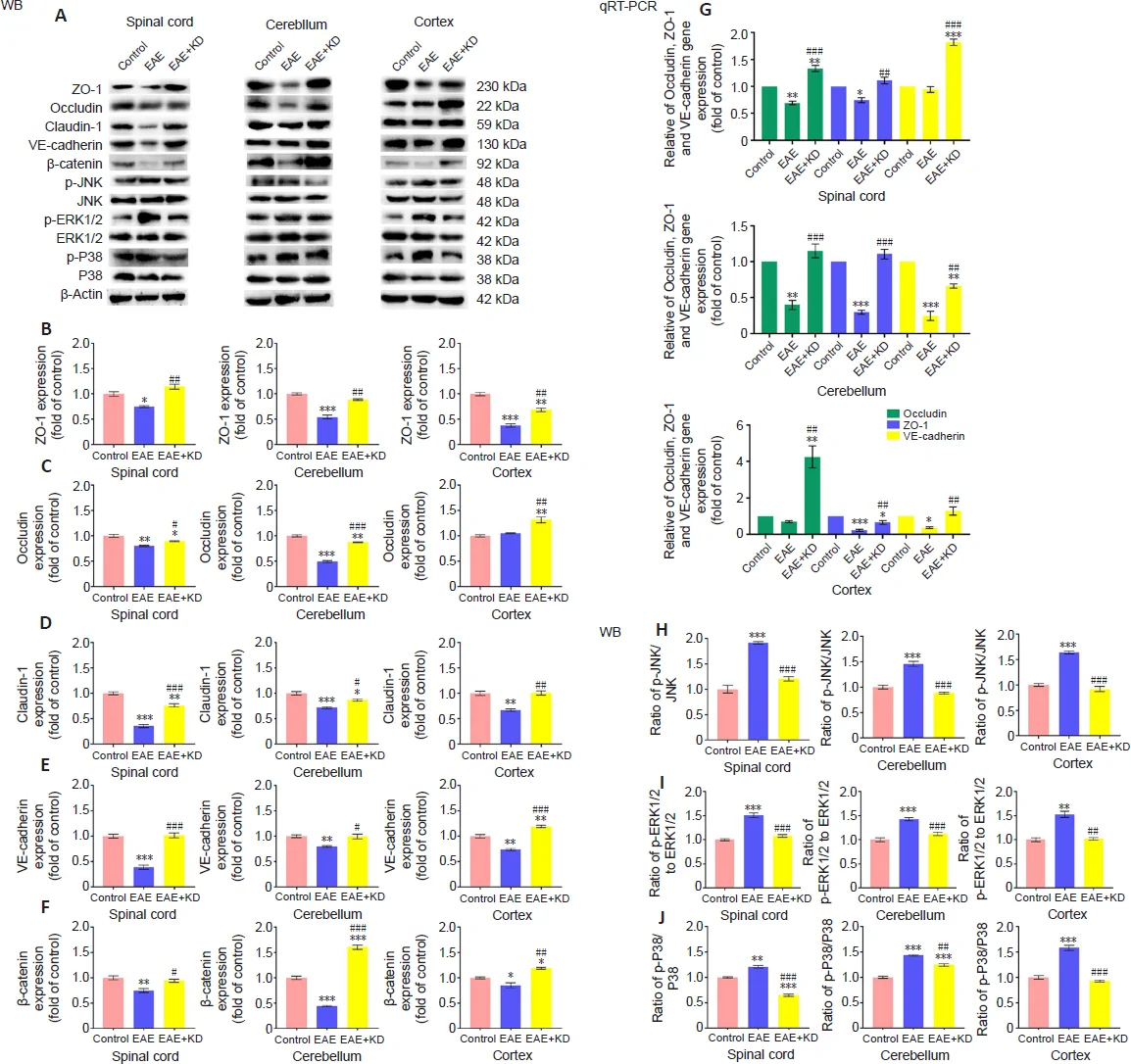
Effects of KD on the expression of ZO-1, occludin, claudin-1, VE-cadherin, β-catenin, p-JNK, JNK, p-ERK1/2, ERK1/2, p-P38 and P38 in the spinal cord, cerebellum and cortex of mice. (A) Representative western blot images of ZO-1, occludin, claudin-1, VE-cadherin, β-catenin, p-JNK, JNK, p-ERK1/2, ERK1/2, p-P38, and P38 in different groups. β-Actin was used as an internal control for normalization. Quantitative analysis of the expression of blood-brain barrier/blood-spinal cord barrier-related proteins, including ZO-1 (B), Occludin (C), Claudin-1 (D), VE-cadherin (E), and β-catenin (F), was performed. (G) qRT-PCR analysis of Occludin, ZO-1 and VE-cadherin mRNA transcripts in the spinal cord, cerebellum and cortex of mice in the different groups. (H) Ratio of p-JNK to JNK. (I) Ratio of p-ERK1/2 to ERK1/2. (J) Ratio of p-P38 to P38. Data are presented as the mean ± SEM (*n* = 4). *P < 0.05, **P < 0.01, ***P < 0.001, *vs.* control group; #*P* < 0.05, ##*P* < 0.01, ###*P* < 0.001, *vs*. EAE group (one-way analysis of variance with Tukey’s *post hoc* test). EAE: Experimental autoimmune encephalomyelitis; KD: ketogenic diet; qRT-PCR: quantitative reverse transcription-polymerase chain reaction.

Western blot analysis (**Figure 4A–F**) demonstrated that, compared with the control group, the EAE group exhibited significantly decreased expression of ZO-1 (spinal cord, *P* < 0.05; cerebellum, *P* < 0.001; cortex, *P* < 0.001), occludin (spinal cord, *P* < 0.01; cerebellum, *P* < 0.001), claudin-1 (spinal cord, *P* < 0.001; cerebellum, *P* < 0.001; cortex, *P* < 0.01), VE-cadherin (spinal cord, *P* < 0.001; cerebellum, *P* < 0.01; cortex, *P* < 0.01), and β-catenin (spinal cord, *P* < 0.01; cerebellum, *P* < 0.001; cortex, *P* < 0.05) across multiple CNS regions. In contrast, KD administration significantly increased the expression levels of all these proteins (ZO-1 [spinal cord, *P* < 0.01; cerebellum, *P* < 0.01; cortex, *P* < 0.01]; occludin [spinal cord, *P* < 0.05; cerebellum, *P* < 0.001; cortex, *P* < 0.01]; claudin-1 [spinal cord, *P* < 0.001; cerebellum, *P* < 0.05; cortex, *P* < 0.01]; VE-cadherin [spinal cord, *P* < 0.001; cerebellum, *P* < 0.05; cortex, *P* < 0.001]; and β-catenin [spinal cord, *P* < 0.05; cerebellum, *P* < 0.001; cortex, *P* < 0.01]) in the EAE + KD group compared with the EAE group.

The RT-PCR results were consistent with these findings. While occludin, ZO-1, and VE-cadherin mRNA levels were strongly downregulated in the spinal cord, cerebellum, and cortex of EAE mice compared with control mice, they were upregulated in the EAE + KD group (occludin [spinal cord, *P* < 0.001; cerebellum; *P* < 0.001; cortex, *P* < 0.01]; ZO-1 [spinal cord, *P* < 0.01; cerebellum, *P* < 0.001; cortex, *P* < 0.01]; VE-cadherin [spinal cord, *P* < 0.001; cerebellum *P* < 0.01; cortex, *P* < 0.01]; **Figure 4G**). These results suggest that the KD may lead to improved BBB and BSCB integrity in EAE mice, preventing BBB and BSCB leakage.

We next investigated the mechanisms responsible for the upregulation of BBB/BSCB-related proteins by the KD. Previous studies showed that Occludin and ZO-1 expressions are modulated by MAPK (JNK, ERK1/2, and p38) signaling (Lan et al., 2019; Guan et al., 2023). Thus, we measured the phosphorylated (P) levels of these factors (P-JNK, P-ERK1/2 and P-p38). Western blot showed downregulated levels of P-JNK, P-ERK1/2 and P-p38 in the EAE + KD group compared with the EAE group; JNK, ERK1/2, and p38 levels were unchanged (**Figure 4A**). Quantitative analysis showed that the EAE group exhibited significantly increased ratios of p-JNK/JNK (spinal cord, *P* < 0.001; cerebellum; *P* < 0.001; cortex, *P* < 0.001), p-ERK1/2/ERK1/2 (spinal cord, *P* < 0.001; cerebellum; *P* < 0.001; cortex, *P* < 0.01), and p-p38/p38 (spinal cord, *P* < 0.01; cerebellum; *P* < 0.001; cortex, *P* < 0.001) compared with the control group. This effect was effectively counteracted by KD administration, which significantly reduced the phosphorylation ratios of all three proteins (p-JNK/JNK [spinal cord, *P* < 0.001; cerebellum; *P* < 0.001; cortex, *P* < 0.001]; p-ERK1/2/ERK1/2 [spinal cord, *P* < 0.001; cerebellum; *P* < 0.01; cortex, *P* < 0.01]; p-p38/p38 [spinal cord, *P* < 0.001; cerebellum; *P* < 0.01; cortex, *P* < 0.001]) in the EAE + KD group compared with the EAE group (**Figure 4H–J**). These results suggested that the KD increased the expression of BBB/BSCB-related proteins, such as Occludin and ZO-1, partly as a result of downregulated MAPK signaling.

### Ketogenic diet administration inhibits activation of astrocytes and microglia in experimental autoimmune encephalomyelitis mice

**Figure 5:**
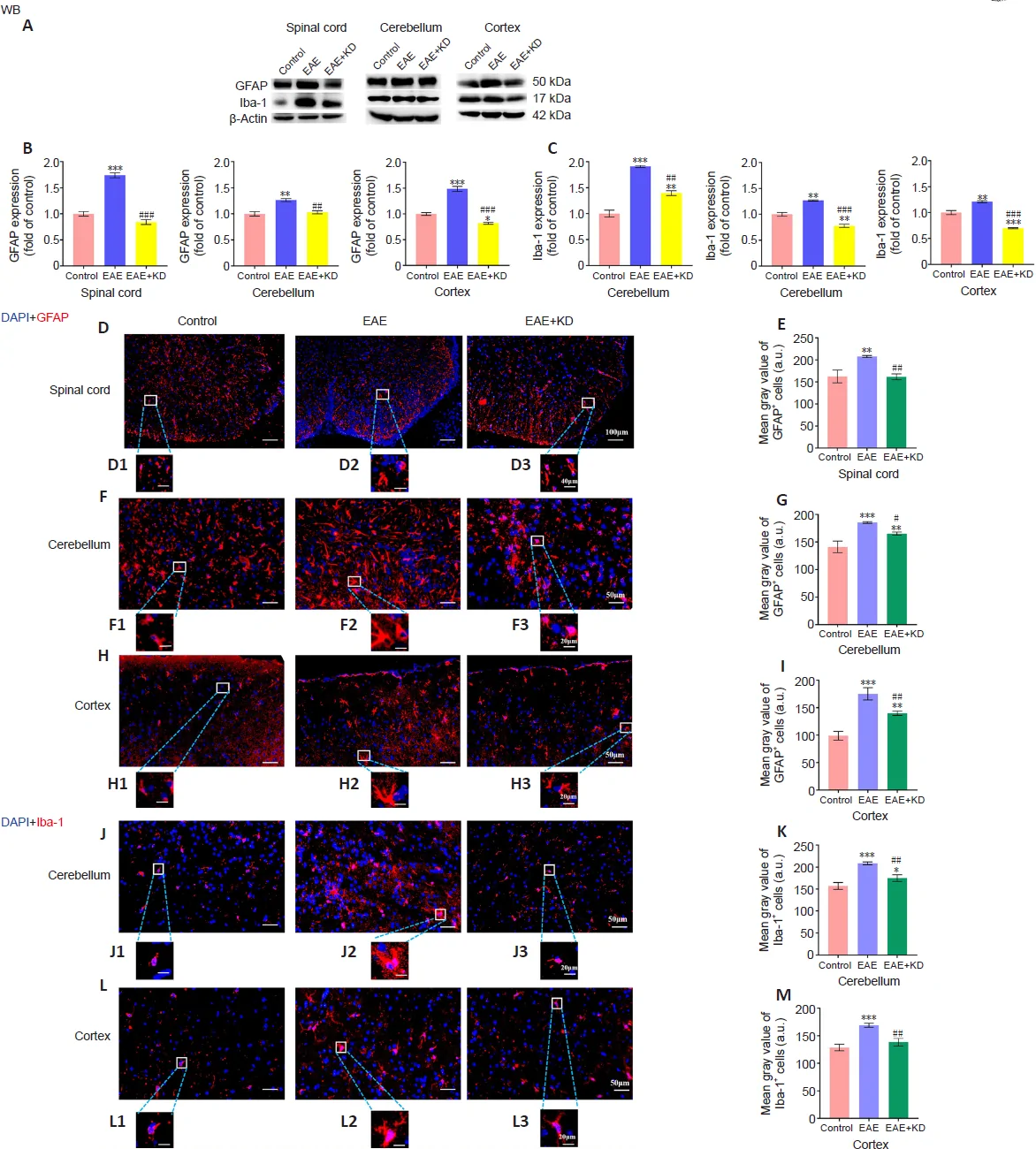
GFAP and Iba-1 immunofluorescence staining of the spinal cord, cerebellum and cortex of the mice. (A) Representative western blot images of GFAP, Iba-1 and β-actin in the different groups. β-actin was used as an internal control for normalization. Quantitative analysis of GFAP (B) and Iba-1 (C). Data are presented as the mean ± SEM (*n* = 4). (D) Representative images of immunofluorescence staining of lesion areas in the spinal cord stained for GFAP in the control, EAE, and EAE + KD groups; scale bars: 100 μm. The white boxes in the images are magnified to show the detailed morphology of GFAP-stained cells in the spinal cord in the control (D1), EAE (D2), and EAE + KD groups (D3); scale bars: 40 μm. (F) Representative images of immunofluorescence staining of the cerebellum for GFAP in the control, EAE and EAE + KD groups; scale bars: 50 μm. The white boxes in the images are magnified to show the detailed morphology of GFAP-stained cells in the cerebellum in the control (F1), EAE (F2), and EAE + KD groups (F3); scale bars: 20 μm. (H) Representative images of immunofluorescence staining of the lesion area in the cortex stained for GFAP in the control, EAE and EAE + KD groups; scale bars: 50 μm. The white boxes in the images are magnified to show the detailed morphology of GFAP-stained cells in the cortex among the control (H1), EAE (H2), and EAE + KD groups (H3); scale bars: 20 μm. (E, G, I) Quantification of GFAP^+^ cells in the spinal cord (E), cerebellum (G), and cortex (I) of the three groups is presented as the relative mean gray value. (J) Representative images of immunofluorescence staining of the lesion area in the cerebellum stained for Iba-1 in the control, EAE and EAE + KD groups; scale bars: 50 μm. The white boxes in the images are magnified to show the detailed morphology of GFAP-stained cells in the cerebellum in the control (J1), EAE (J2), and EAE + KD groups (J3); scale bars: 20 μm. (L) Representative images of immunofluorescence staining of the lesion area in the cortex stained for Iba-1 in the control, EAE and EAE + KD groups; scale bars: 50 μm. The white boxes in the images are magnified to show the detailed morphology of GFAP-stained cells in the cortex among the control (L1), EAE (L2), and EAE + KD groups (L3); scale bars: 20 μm. (K, M) Quantification of Iba-1^+^ cells in the cerebellum (K) and cortex (M) of the three groups is presented as the relative mean gray value. Data are presented as the mean ± SEM (*n* = 4). *P < 0.05, **P < 0.01, ***P < 0.001, *vs*. control group; #*P* < 0.05, ##*P* < 0.01, ###*P* < 0.001, *vs*. EAE group (one-way analysis of variance with Tukey’s *post hoc* test). EAE: Experimental autoimmune encephalomyelitis; GFAP: glial fibrillary acidic protein; Iba-1: ionized calcium-binding adapter molecule 1; KD: ketogenic diet.

Given the established role of glial cells in MS pathogenesis (Yong, 2022), we investigated the effects of KD on astrocyte and microglia activation. Western blot analysis demonstrated an upregulation of GFAP (spinal cord, *P* < 0.001; cerebellum; *P* < 0.01; cortex, *P* < 0.001) and Iba-1 (spinal cord, *P* < 0.001; cerebellum; *P* < 0.01; cortex, *P* < 0.01) in the EAE group *versus* controls, reflecting glial cell activation. Conversely, KD administration significantly reduced the expression of GFAP (spinal cord, *P* < 0.001; cerebellum; *P* < 0.01; cortex, *P* < 0.001) and Iba-1 (spinal cord, *P* < 0.01; cerebellum; *P* < 0.001; cortex, *P* < 0.001) in the EAE + KD group compared with the EAE group across all examined CNS regions (**Figure 5A–C**). Immunofluorescence analysis of GFAP in CNS regions revealed distinct morphological alterations in EAE mice, characterized by hypertrophic somata and thickened, less branched processes, which are hallmarks of astrocyte activation (**Figure 5D2**, **F2**, and **H2**). KD administration markedly attenuated these changes, restoring more quiescent morphologies with smaller cell bodies and finer processes (**Figure 5D3**, **5F3**, and **5H3**). Quantitative analysis confirmed reduced GFAP expression in EAE + KD mice compared with EAE mice (spinal cord; *P* < 0.01, cerebellum; *P* < 0.05; cortex, *P* < 0.01; **Figure 5E**, **G**, and **I**, respectively), demonstrating KD-mediated suppression of astrocyte reactivity induced by EAE. The effect of the KD on microglia was assessed by Iba-1 immunostaining. As shown in **Figure 5J** and **L**, the microglia morphology in control mice showed a small cell body and thin processes. In contrast, the morphology of activated microglia in EAE mice was characterized by an enlarged soma and shortened cell processes (**Figure 5J2** and **L2**). In EAE + KD mice, the microglia displayed ramified processes with a small cell body and several long-branched processes (**Figure 5J3** and **L3**). Immunofluorescence staining showed a significant increase in Iba-1+ (cerebellum, *P* < 0.001, cortex, *P* < 0.001) expression in the EAE group relative to controls. Conversely, KD administration effectively suppressed this microglial response, leading to a significant reduction in Iba-1^+^ expression in the EAE + KD group compared with the EAE group in the cerebellum (*P* < 0.01) and cortex (*P* < 0.01) (**Figure 5K** and **M**). These findings are in line with our previous findings showing that KD administration diminished the spinal level of Iba-1^+^ (Sun et al., 2023). These results suggested that the KD suppresses EAE-induced microgliosis. Taken together, these data suggested that KD decreases the activation of microglia and astrocytes in EAE mice.

### The ketogenic diet inhibits A1 type astrocytes and promotes transformation to the A2 astrocyte phenotype in experimental autoimmune encephalomyelitis mice

We next investigated the impact of the KD on astrocyte and microglia phenotype in EAE mice. Reactive astrocytes are categorized into two functionally distinct subsets: the neurotoxic A1 phenotype (characterized by elevated expression of complement components including Amigo2, C3, and GBP2) and neuroprotective A2 phenotype (marked by increased SPHK1, PTX3, and S100a10 levels). These molecular signatures are reliable biomarkers for astrocyte subtyping (Fan and Huo, 2021; Une et al., 2021; Luchena et al., 2022).

**Figure 6:**
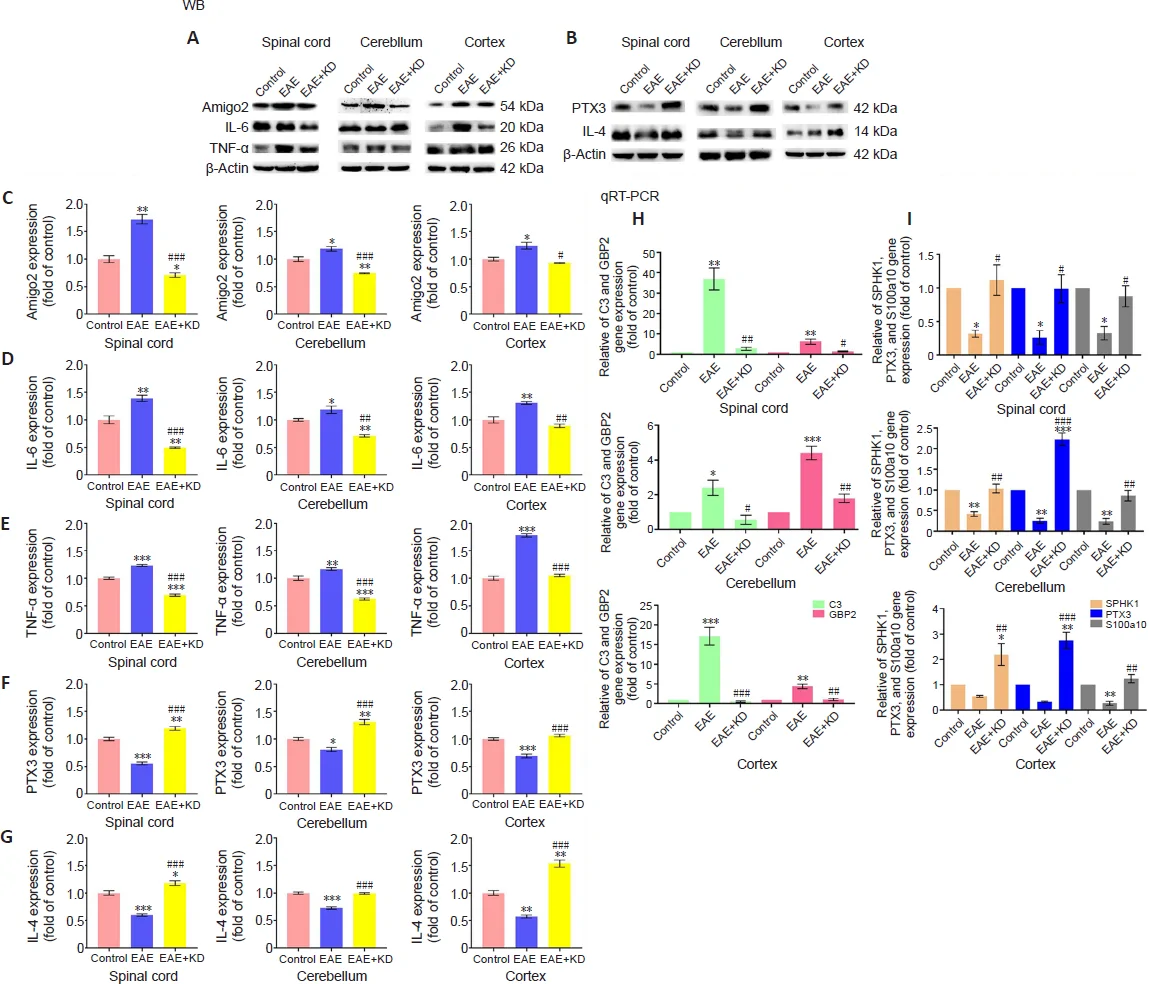
KD administration suppresses A1-related markers (Amigo2, C3, and GBP2), IL-6 and TNF-α and promotes A2-related markers (PTX3, SPHK1, and S100a10) and IL-4 in the spinal cord, cerebellum and cortex of EAE mice. (A) Representative western blot images of Amigo2, IL-6, and TNF-α. (B) Representative western blot images of PTX3 and IL-4. β-actin was used as the internal control for normalization. (C–E) Quantitative analysis of A1-related marker (Amigo2) (C), IL-6 (D), and TNF-α expression (E) in the spinal cord, cerebellum and cortex. (F, G) Quantitative analysis of A2-related marker (PTX3) (F) and IL-4 expression (G) in the spinal cord, cerebellum and cortex. (H) Quantitative analysis of the expression of A1-related genes, including C3 and GBP2, in the spinal cord, cerebellum, and cortex. (I) Quantitative analysis of the expression of A2-related genes, including SPHK1, PTX3 and S100a10, in the spinal cord, cerebellum and cortex. Data are presented as the mean ± SEM (*n* = 4). *P < 0.05, **P < 0.01, ***P < 0.001, *vs.* control group; #*P* < 0.05, ##*P* < 0.01, ###*P* < 0.001, *vs*. EAE group (one-way analysis of variance with Tukey’s *post hoc* test). EAE: Experimental autoimmune encephalomyelitis; IL: interleukin; KD: ketogenic diet; qRT-PCR: quantitative reverse transcription-polymerase chain reaction; TNF-α: tumor necrosis factor-α.

To evaluate the effects of the KD on astrocyte polarization, we analyzed A1/A2-specific markers in CNS tissues. Western blot analysis revealed that, compared with the control group, EAE mice exhibited elevated expression of the A1 astrocyte marker Amigo2 (spinal cord; *P* < 0.01, cerebellum; *P* < 0.05; cortex, *P* < 0.05) and the pro-inflammatory cytokines IL-6 (spinal cord; *P* < 0.01, cerebellum; *P* < 0.05; cortex, *P* < 0.01) and TNF-α (spinal cord; *P* < 0.001, cerebellum; *P* < 0.01; cortex, *P* < 0.001), whereas the A2 marker PTX3 (spinal cord; *P* < 0.001, cerebellum; *P* < 0.05; cortex, *P* < 0.001) and the anti-inflammatory cytokine IL-4 (spinal cord; *P* < 0.001, cerebellum; *P* < 0.001; cortex, *P* < 0.01) were reduced. KD administration significantly counteracted these changes, decreasing Amigo2 (spinal cord, *P* < 0.001; cerebellum, *P* < 0.001; cortex, *P* < 0.05), IL-6 (spinal cord; *P* < 0.001, cerebellum; *P* < 0.01; cortex, *P* < 0.01) and TNF-α (spinal cord; *P* < 0.001, cerebellum; *P* < 0.001; cortex, *P* < 0.001), while upregulating PTX3 (spinal cord; *P* < 0.001, cerebellum; *P* < 0.001; cortex, *P* < 0.001) and IL-4 (spinal cord; *P* < 0.001, cerebellum; *P* < 0.001; cortex, *P* < 0.001) in EAE + KD mice compared with the EAE group (**Figure 6A–G**). Consistent with these protein-level findings, RT-PCR results showed that, relative to the EAE group, the EAE + KD group displayed downregulated mRNA levels of C3 (spinal cord; *P* < 0.01, cerebellum; *P* < 0.05; cortex, *P* < 0.001) and GBP2 (spinal cord; *P* < 0.05, cerebellum; *P* < 0.01; cortex, *P* < 0.01), and enhanced expression of SPHK1 (spinal cord; *P* < 0.05, cerebellum; *P* < 0.01; cortex, *P* < 0.01), PTX3 (spinal cord; *P* < 0.05, cerebellum; *P* < 0.001; cortex, *P* < 0.001), and S100a10 (spinal cord; *P* < 0.05, cerebellum; *P* < 0.01; cortex, *P* < 0.01) (**Figure 6H** and **I**).

**Figure 7:**
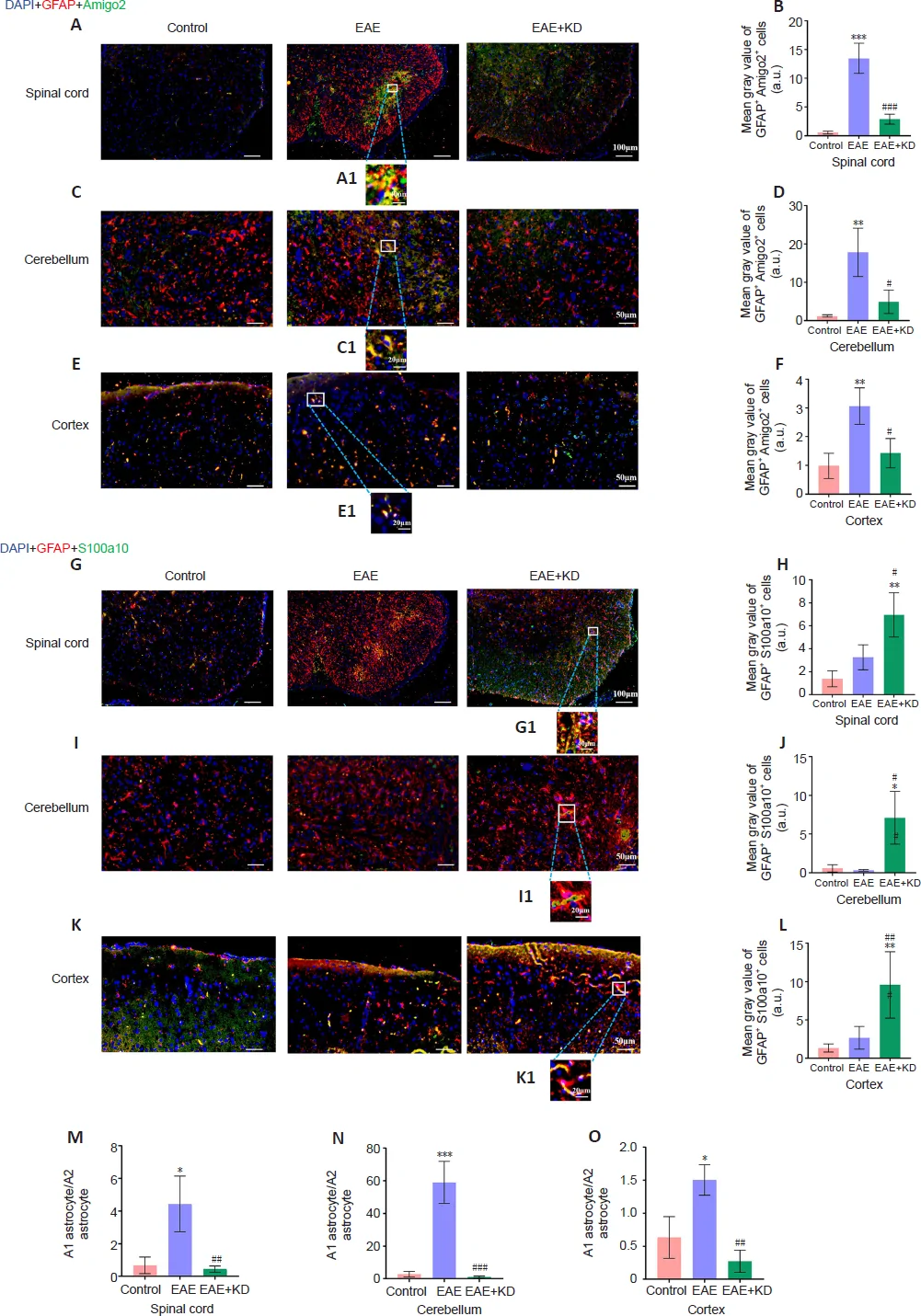
Immunofluorescent double staining for GFAP^+^/Amigo2^+^ cells and GFAP^+^/S100a10^+^ cells in mice. (A, C, E) Representative images of double immunofluorescence staining of GFAP^+^/Amigo2^+^ cells in the spinal cord (A), cerebellum (C), and cortex (E). White box inset: magnification of selected images of GFAP^+^ (red)/Amigo2^+^ (green) double-stained cells in the spinal cord (A1), cerebellum (C1), and cortex (E1). (B, D, F) Quantification of GFAP^+^/Amigo2^+^ cells in the spinal cord (B), cerebellum (D), and cortex (F) of the three groups is presented as the relative mean gray value. (G, I, K) Representative images of double immunofluorescence staining of GFAP^+^/S100a10^+^ cells in the spinal cord (G), cerebellum (I) and cortex (K). White box inset: magnification of the selected images of GFAP^+^ (red)/S100a10^+^ (green) double-stained cells in the spinal cord (G1), cerebellum (I1), and cortex (K1). (H, J, L) Quantification of GFAP^+^/S100a10^+^ cells in the spinal cord (H), cerebellum (J), and cortex (L) of the three groups is presented as the relative mean gray value. Scale bars: 100 μm in A, G; 40 μm in A1 and G1; 50 μm in C, E, I and K; 20 μm in C1, E1, I1, and K1. *P < 0.05, **P < 0.01, ***P < 0.001, *vs.* control group; #*P* < 0.05, ##*P* < 0.01, ###*P* < 0.001, *vs*. EAE group (one-way analysis of variance with Tukey’s *post hoc* test). EAE: Experimental autoimmune encephalomyelitis; GFAP: glial fibrillary acidic protein; KD: ketogenic diet.

Immunofluorescence co-staining of Amigo2/GFAP and S100a10/GFAP confirmed these findings (**Figure 7A**, **C**, and **E**). EAE mice exhibited increased GFAP^+^/Amigo2^+^ signals across CNS regions, which KD administration markedly suppressed (spinal cord, *P* < 0.001; cerebellum, *P* < 0.05; cortex, *P* < 0.05; **Figure 7B**, **D**, and **F**, respectively). Conversely, GFAP^+^/S100a10^+^ (**Figure 7G**, **I** and **K**) co-localization was elevated in KD-fed mice (spinal cord, *P* < 0.05; cerebellum, *P* < 0.01; cortex, *P* < 0.01; **Figure 7H**, **J**, and **L**, respectively). These results demonstrate the dual effect of the KD in inhibiting A1 polariza tion and promoting A2 activation.

Taken together, these data suggested that the KD may alter the phenotypic state of astrocytes in EAE mice.

### The ketogenic diet suppresses M1 type microglia and promotes the M2 phenotype in experimental autoimmune encephalomyelitis mice

We next examined whether the KD affects the phenotype of microglial cells. Transcriptomic studies classified reactive microglia into two subsets analogous to astrocyte phenotypes: M1 (pro-inflammatory) and M2 (anti-inflammatory) (Sun et al., 2023).

**Figure 8:**
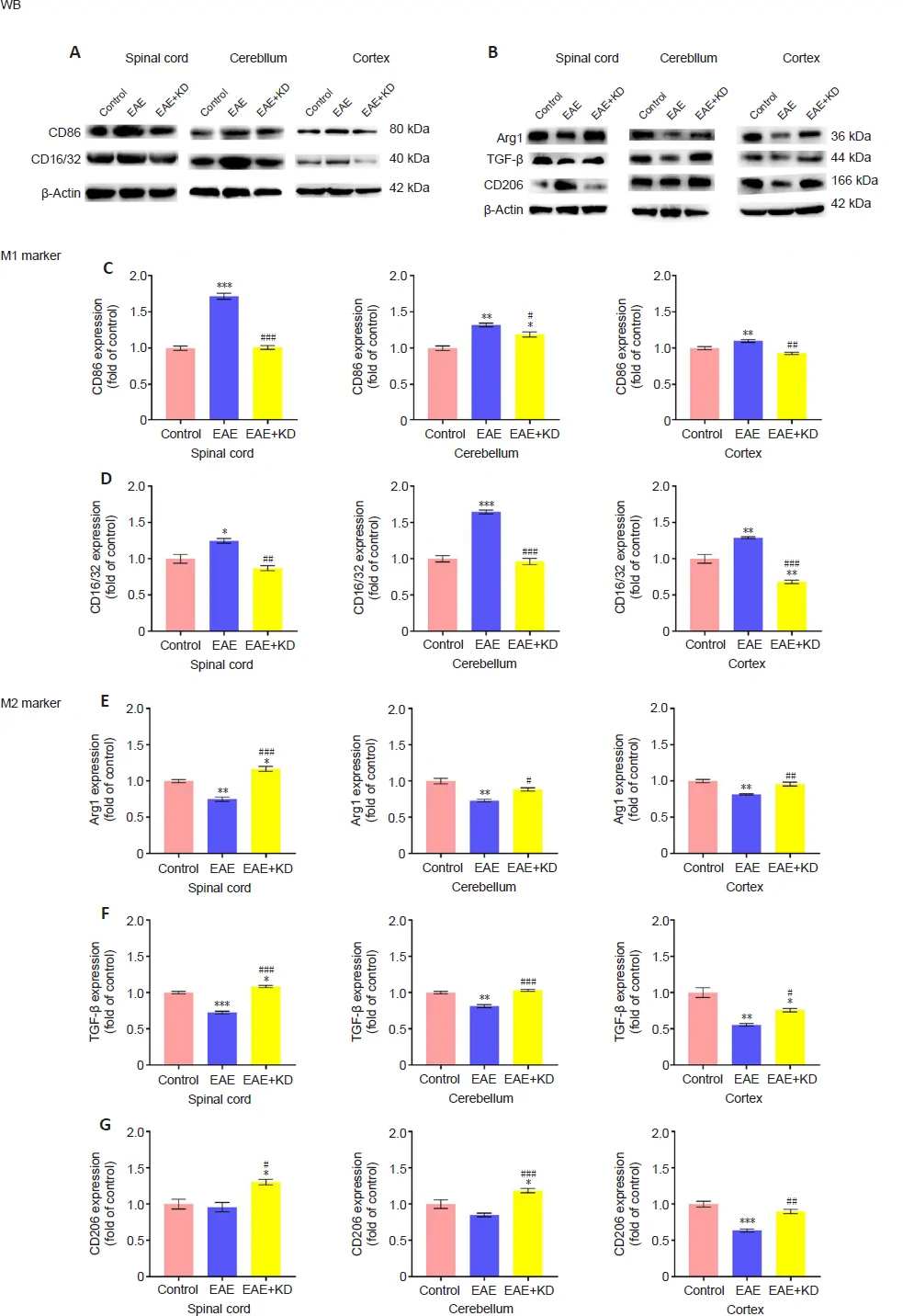
KD suppresses M1-related marker expression and promotes M2-related marker expression in the spinal cord, cerebellum and cortex of mice. (A) Representative western blot images of CD86 and CD16/32 expression. (B) Representative western blot images of Arg1, TGF-β, and CD206 expression. β-Actin was used as the internal control for normalization. (C, D) Quantitative analysis of the expression of M1 markers, including CD86 (C) and CD16/32 (D), in the spinal cord, cerebellum and cortex. (E, F, G) Quantitative analysis of the expression of M2 markers, including Arg1 (E), TGF-β (F) and CD206 (G), in the spinal cord, cerebellum and cortex. Data are presented as the mean ± SEM (*n* = 4). *P < 0.05, **P < 0.01, ***P < 0.001, *vs.* control group; #*P* < 0.05, ##*P* < 0.01, ###*P* < 0.001, *vs*. EAE group (one-way analysis of variance with Tukey’s *post hoc* test). EAE: Experimental autoimmune encephalomyelitis; KD: ketogenic diet; TGF-β: transforming growth factor-β.

Western blot analysis revealed that EAE mice showed upregulated M1 markers (CD86 [spinal cord, *P* < 0.001; cerebellum, *P* < 0.01; cortex, *P* < 0.01] and CD16/32 [spinal cord, *P* < 0.05; cerebellum, *P* < 0.001; cortex, *P* < 0.01]) and downregulation of M2 markers (Arg1[spinal cord, *P* < 0.01; cerebellum, *P* < 0.01; cortex, *P* < 0.01], transforming growth factor-β (TGF-β) [spinal cord, *P* < 0.001; cerebellum, *P* < 0.01; cortex, *P* < 0.01], and CD206 [cortex, *P* < 0.001]) compared with the control group. KD administration effectively reversed this microglial polarization pattern, significantly downregulating M1 markers (CD86 [spinal cord, *P* < 0.001; cerebellum, *P* < 0.05; cortex, *P* < 0.01] and CD16/32 [spinal cord, *P* < 0.01; cerebellum, *P* < 0.001; cortex, *P* < 0.001]) and upregulating M2 markers (Arg1[spinal cord, P < 0.001; cerebellum, *P* < 0.05; cortex, *P* < 0.01], TGF-β [spinal cord, *P* < 0.001; cerebellum, *P* < 0.001; cortex, *P* < 0.05], and CD206 [spinal cord, *P* < 0.05; cerebellum, *P* < 0.01; cortex, *P* < 0.01]) in the EAE + KD group compared with the EAE group (**Figure 8A–G**).

**Figure 9:**
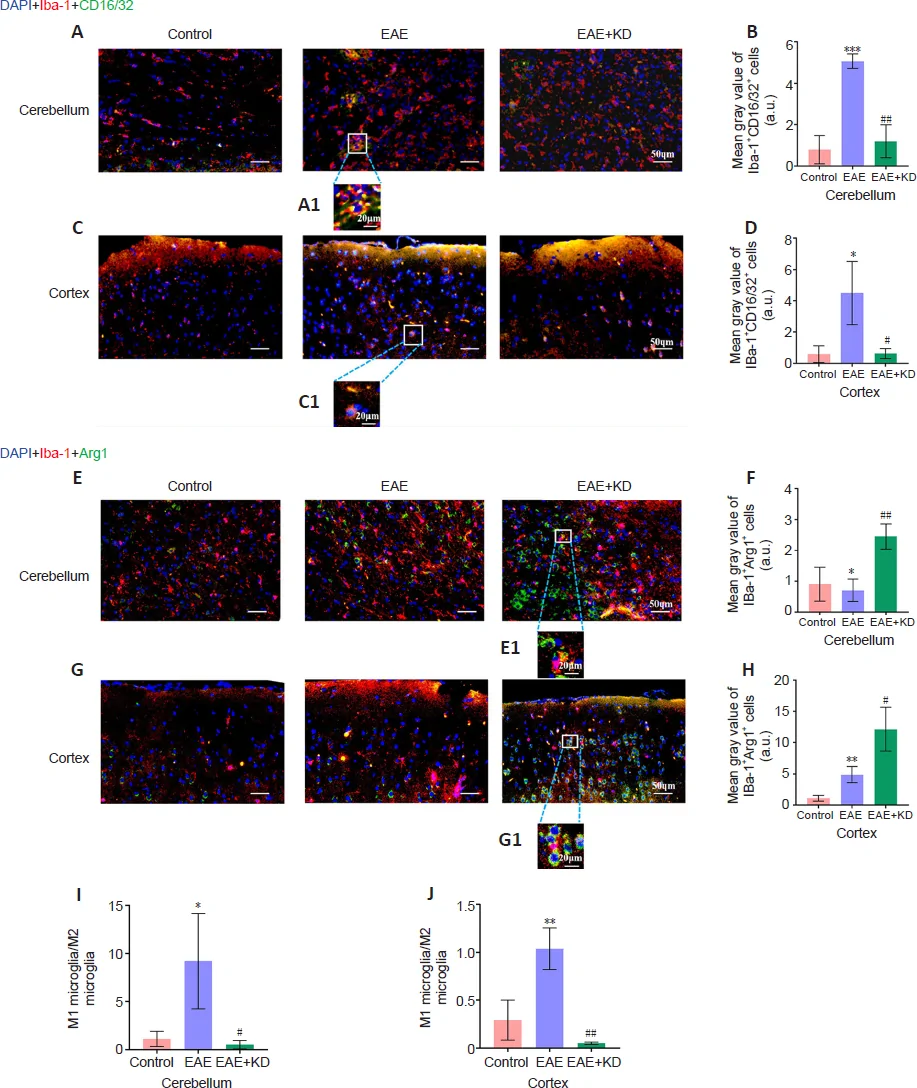
Immunofluorescence double-staining of microglia in mice revealed Iba-1^+^/CD16/32^+^ cells (proinflammatory phenotype) and Iba-1^+^/Arg1^+^ cells (anti-inflammatory phenotype). (A, C) Representative images of double immunofluorescence staining of Iba-1^+^/CD16/32^+^ cells in the cerebellum (A) and cortex (C). White box inset: magnification of selected images of Iba-1^+^ (red)/CD16/32^+^ (green) double-stained cells in the cerebellum (A1) and cortex (C1). (B, D) Quantification of Iba-1^+^/CD16/32^+^ cells in the cerebellum (B) and cortex (D) of the three groups is presented as the relative mean gray value. (E, G) Representative images of double immunofluorescence staining of Iba-1^+^/Arg1^+^ cells in the cerebellum (E) and cortex (G). White box inset: magnification of selected images of Iba-1^+^ (red)/Arg1^+^ (green) double-stained cells in the cerebellum (E1) and cortex (G1). (F, H) Quantification of Iba-1^+^/Arg1^+^ cells in the cerebellum (F) and cortex (H) of the three groups is presented as the relative mean gray value. Scale bars: 50 μm in A, C, E and G; 50 μm in A1, C1, E1, and G. (I, J) Quantification of the M1/M2 ratios in the cerebellum and cortex of the three groups. Data are presented as the means ± SEMs (*n* = 4). *P < 0.05, **P < 0.01, ***P < 0.001, *vs.* control group; #*P* < 0.05, ##*P* < 0.01, ###*P* < 0.001, *vs*. EAE group (one-way analysis of variance with Tukey’s *post hoc* test). EAE: Experimental autoimmune encephalomyelitis; KD: ketogenic diet; Iba-1: ionized calcium-binding adapter molecule 1.

Consistent with the Western blot findings, double-labeling immunofluorescence across the spinal cord, cerebellum, and cortex revealed that, compared with the control group, EAE mice exhibited a significant increase in Iba1^+^/CD16/32^+^ (M1) cells (cerebellum, *P* < 0.001; cortex, *P* < 0.05) and a decrease in Iba1^+^/Arg1^+^ (M2) cells (cerebellum, *P* < 0.05; cortex, *P* < 0.01). KD administration effectively reversed this microglial polarization, significantly reducing M1 cell (Iba1^+^/CD16/32^+^, cerebellum, *P* < 0.01; cortex, *P* < 0.05) abundance and increasing M2 cell (Iba1^+^/Arg1^+^, cerebellum, *P* < 0.01; cortex, *P* < 0.05) abundance in the EAE + KD group compared with the EAE group (**Figure 9A–H**). Moreover, M1/M2 microglial polarization ratio was significantly elevated across the cerebellum (M1/M2 ratio, *P* < 0.05) and cortex (M1/M2 ratio, *P* < 0.01) in EAE mice compared with the control group, and this shift was markedly reversed by KD administration, which significantly reduced the ratio in EAE + KD mice relative to the EAE group (M1/M2 ratio, cerebellum, *P* < 0.05; cortex, *P* < 0.01; **Figure 9I** and **J**).

These results demonstrate that EAE activates M1 microglia, while the KD shifts the balance toward neuroprotective M2 polarization. Previous studies demonstrated that M1 microglia induce the activation of A1 astrocytes, indicating a feedback loop between astrocytes and microglia (Guo et al., 2022).

### NOD-, LRR- and pyrin domain-containing protein 3, JAK2/STAT3, nuclear factor kappa B, and PI3K/AKT signaling pathways are involved in ketogenic diet-induced astrocyte polarization

We further explored the mechanisms by which the KD drives astrocyte phenotype transformation. Emerging evidence has implicated the NLRP3 inflammasome, JAK2/STAT3, nuclear factor kappa B (NF-κB), and PI3K/AKT pathways in astrocyte A1 polarization (Hou et al., 2020; Wang et al., 2021; Li et al., 2022b). HDAC3 is a transcriptional activator of NLRP3, NF-κB, and JAK2 (Niu et al., 2022; Shen et al., 2022; Sun et al., 2023). We therefore examined the effects of KD on these pathways.

**Figure 10:**
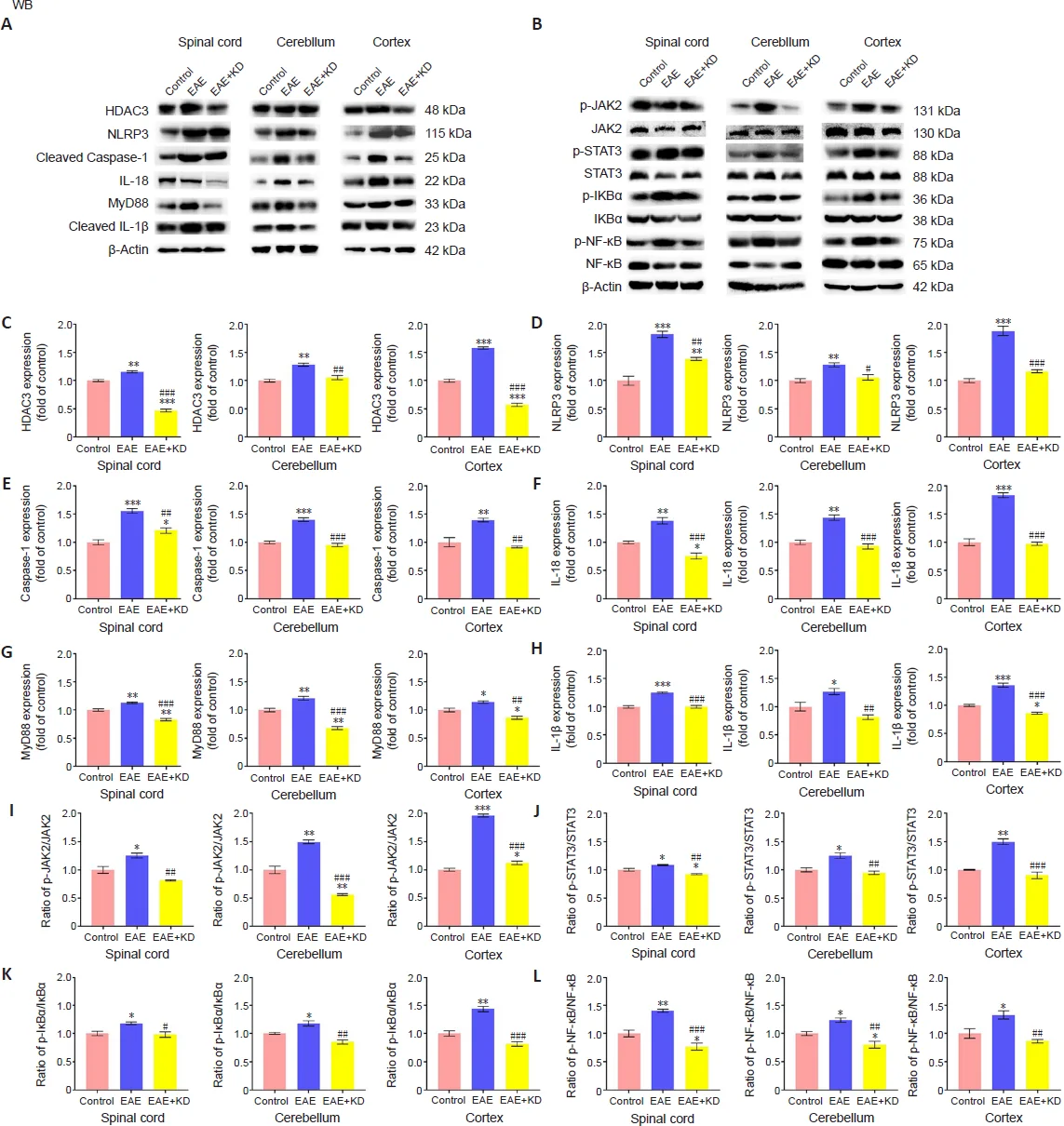
Effects of KD on the expression of HDAC3, NLRP3, caspase-1, IL-18, MyD88, and IL-1β and the ratios of p-JAK2/JAK2, p-STAT3/STAT3, p-IKBα/IKBα and p-NF-κB/NF-κB in the spinal cord, cerebellum and cortex of mice. (A) Representative WB images of HDAC3, NLRP3, caspase-1, IL-18, MyD88, and IL-1β. β-actin was used as the internal control for normalization. (B) Representative WB images of p-JAK2, JAK2, p-STAT3, STAT3, p-IKBα, IKBα, p-NF-κB, and NF-κB. β-actin was used as the internal control for normalization. (C–H) Quantitative analysis of the protein expression of HDAC3 (C), NLRP3 (D), caspase-1 (E), IL-18 (F), MyD88 (G), and IL-1β (H) in the spinal cord, cerebellum and cortex. (I–L) Ratios of p-JAK2/JAK2 (I), p-STAT3/STAT3 (J), p-IKBα/IKBα (K), and p-NF-κB/NF-κB (L) in the spinal cord, cerebellum and cortex. Data are presented as the mean ± SEM (*n* = 4). *P < 0.05, **P < 0.01, ***P < 0.001, *vs.* control group; #*P* < 0.05, ##*P* < 0.01, ###*P* < 0.001, *vs.* EAE group (one-way analysis of variance with Tukey’s *post hoc* test). EAE: Experimental autoimmune encephalomyelitis; KD: ketogenic diet.

Compared with the control group, EAE mice exhibited significant upregulation of HDAC3 (spinal cord, *P* < 0.01; cerebellum, *P* < 0.01; cortex, *P* < 0.001), NLRP3 (spinal cord, *P* < 0.001; cerebellum, *P* < 0.01; cortex, *P* < 0.001), caspase-1 (spinal cord, *P* < 0.001; cerebellum, *P* < 0.001; cortex, *P* < 0.01), IL-18 (spinal cord, *P* < 0.01; cerebellum, *P* < 0.01; cortex, *P* < 0.001), and downstream effectors (MyD88 [spinal cord, *P* < 0.01; cerebellum, *P* < 0.01; cortex, *P* < 0.05] and IL-1β [spinal cord, *P* < 0.05; cerebellum, *P* < 0.01; cortex, *P* < 0.001]), and increased phosphorylation ratios of JAK2 (spinal cord, *P* < 0.05; cerebellum, *P* < 0.01; cortex, *P* < 0.001), STAT3 (spinal cord, *P* < 0.05; cerebellum, *P* < 0.05; cortex, *P* < 0.01), IKBα (spinal cord, *P* < 0.05; cerebellum, *P* < 0.05; cortex, *P* < 0.01), and NF-κB (spinal cord, *P* < 0.01; cerebellum, *P* < 0.05; cortex, *P* < 0.05), while total protein levels remained unaltered. KD administration effectively reversed these changes, significantly reducing the expression of these inflammatory mediators (HDAC3 [spinal cord, *P* < 0.001; cerebellum, *P* < 0.01; cortex, *P* < 0.001]; NLRP [spinal cord, *P* < 0.01; cerebellum, *P* < 0.05; cortex, *P* < 0.001]; caspase-1 [spinal cord, *P* < 0.01; cerebellum, *P* < 0.001; cortex, *P* < 0.01]; IL-18 [spinal cord, *P* < 0.001; cerebellum, *P* < 0.001; cortex, *P* < 0.001]; MyD88 [spinal cord, *P* < 0.001; cerebellum, *P* < 0.001; cortex, *P* < 0.01]; IL-1β [spinal cord, *P* < 0.001; cerebellum, *P* < 0.01; cortex, *P* < 0.001]) and the phosphorylation ratios (p-JAK2/JAK2 [spinal cord, *P* < 0.01; cerebellum, *P* < 0.001; cortex, *P* < 0.001]; p-STAT3/STAT3 [spinal cord, *P* < 0.01; cerebellum, *P* < 0.01; cortex, *P* < 0.001]; p-IKBα/IKBα [spinal cord, *P* < 0.05; cerebellum, *P* < 0.01; cortex, *P* < 0.001]; p-NF-κB/NF-κB [spinal cord, *P* < 0.001; cerebellum, *P* < 0.01; cortex, *P* < 0.01]) in EAE + KD mice compared with the EAE group (**Figure 10**). These results suggest that the KD may mediate suppression of A1 polarization by decreasing the expression of HDAC3 and downregulating the activation of NF-κB, NLRP3, and JAK2/STAT3 signaling pathways.

**Figure 11:**
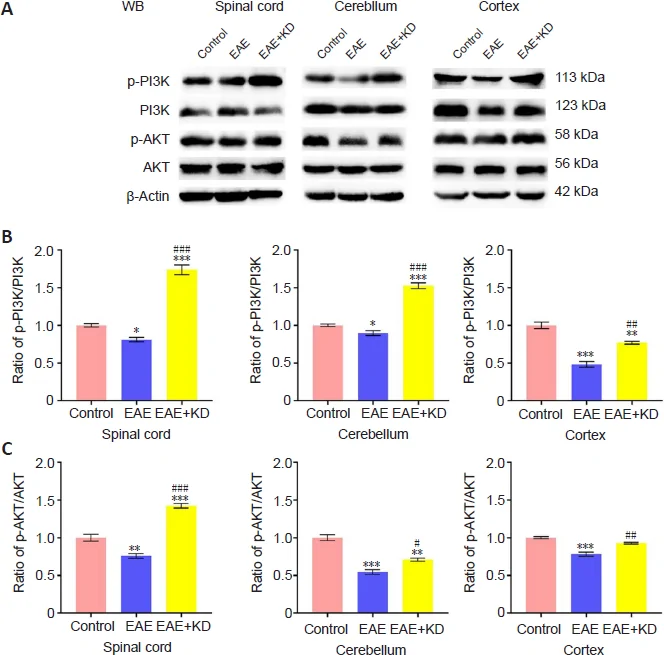
Effects of KD on the activation of the PI3K/AKT signaling pathway in the spinal cord, cerebellum and cortex of mice. (A) Representative WB images of p-PI3K, PI3K, p-AKT, and AKT. β-Actin was used as the internal control for normalization. (B, C) Ratios of p-PI3K/PI3K (B) and p-AKT/AKT (C) in the spinal cord, cerebellum and cortex. Data are presented as the mean ± SEM (*n* = 4). *P < 0.05, **P < 0.01, ***P < 0.001, *vs.* control group; #*P* < 0.05, ##*P* < 0.01, ###*P* < 0.001, *vs.* EAE group (one-way analysis of variance with Tukey’s *post hoc* test). EAE: Experimental autoimmune encephalomyelitis; KD: ketogenic diet; WB: western blotting.

The polarization of astrocytes towards to the A2 phenotype is driven by activation of the PI3K/AKT pathway (Pang et al., 2023). We evaluated GFAP^+^/PI3K^+^ and GFAP^+^/AKT^+^ cells by immunofluorescence double-labeling and PI3K, AKT, P-PI3K, and P-AKT levels by western blot. KD administration significantly increased EAE-induced PI3K and AKT expression in astrocytes (PI3K [spinal cord, *P* < 0.05, cerebellum, *P* < 0.01, and cortex, *P* < 0.001], AKT [spinal cord, *P* < 0.01, cerebellum, *P* < 0.01, and cortex, *P* < 0.001]; **Additional Figure 2**). p-PI3K and p-AKT levels were increased in the spinal cord, cerebellum, and cortex of EAE + KD mice compared with EAE mice (**Figure 11A**). Quantitative analysis revealed significantly elevated p-PI3K/PI3K (spinal cord/cerebellum, *P* < 0.001, and cortex, *P* < 0.01; **Figure 11B**) and P-AKT/AKT ratios (spinal cord, *P* < 0.001, cerebellum, *P* < 0.05, and cortex, *P* < 0.01; **Figure 11C**) in KD-treated EAE mice compared with EAE mice. This enhanced PI3K/AKT pathway activation correlated with increased A2 marker expression, suggesting KD may promote A2 astrocyte polarization through this signaling axis.

### Ketogenic diet administration inhibits activation of the NOD-, LRR- and pyrin domain-containing protein 3 inflammasome in astrocytes

**Figure 12:**
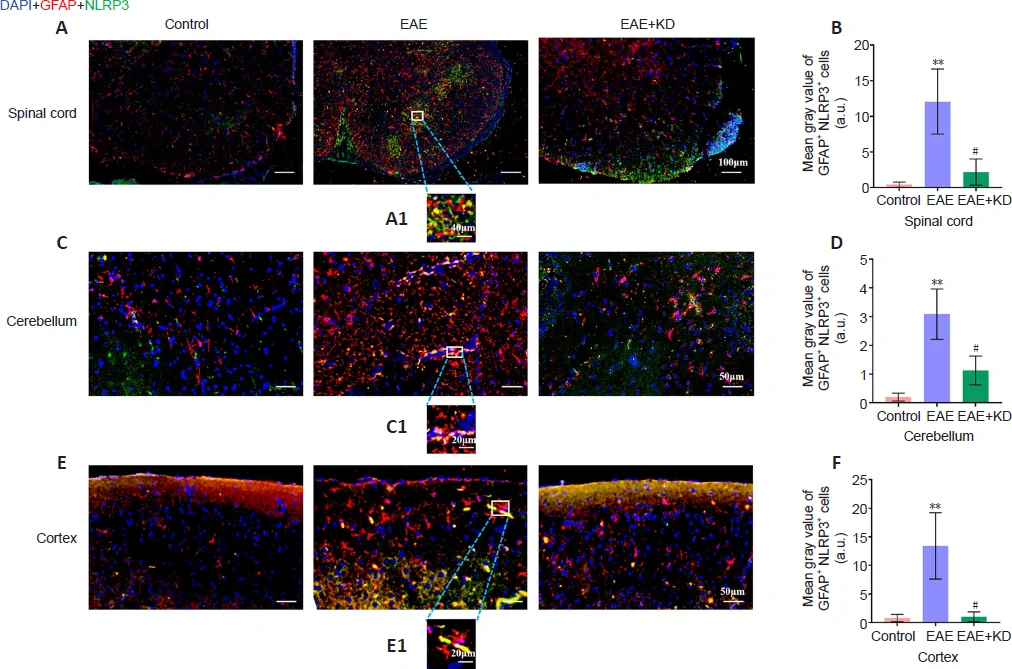
KD inhibits NLRP3 inflammasome expression in the astrocytes of EAE mice. (A, C, E) Double immunofluorescence of GFAP^+^/NLRP3^+^ cells in the spinal cord (A), cerebellum (C), and cortex (E) of the three groups. The white box in the inset shows the colocalization (orange color) of GFAP^+^ (red)/NLRP3^+^ (green) cells in the spinal cord (A1), cerebellum (C1), and cortex (E1). Scale bars: 100 μm in A; 50 μm in C and E; 40 μm in A1; 20 μm in C1 and E1. (B, D, F) Quantification of GFAP^+^/NLRP3^+^ cells in the spinal cord (B), cerebellum (D), and cortex (F) of the three groups is presented as the relative mean gray value. Data are presented as the mean ± SEM (*n* = 4). **P < 0.01, *vs*. control group; #*P* < 0.05, *vs.* EAE group (one-way analysis of variance with Tukey’s *post hoc* test). EAE: Experimental autoimmune encephalomyelitis; GFAP: glial fibrillary acidic protein; KD: ketogenic diet; NLRP3: NOD-, LRR- and pyrin domain-containing protein 3.

Immunofluorescence co-localization analysis demonstrated NLRP3^+^/GFAP^+^ astrocytes in CNS tissues of EAE mice (**Figure 12A**, **C**, and **E**). Quantitative analysis revealed that KD administration EAE mice exhibited significantly elevated NLRP3 expression in GFAP^+^ astrocytes compared with the control group (spinal cord, *P* < 0.01; cerebellum, *P* < 0.01; cortex, *P* < 0.01), an effect that was markedly attenuated by KD administration, which significantly reduced NLRP3 levels in GFAP^+^ cells in EAE + KD mice relative to the EAE group (spinal cord, *P* < 0.05; cerebellum, *P* < 0.05; cortex, *P* < 0.05; **Figure 12B**, **D**, and **F**, respectively), indicating KD-mediated suppression of astrocytic NLRP3 inflammasome activation in EAE mice.

### The ketogenic diet decreases matrix metalloproteinase-2 and -9 expressions and increases tissue inhibitor of metalloproteinase-1 and -2 expressions in the spinal cord, cerebellum, and cortex of experimental autoimmune encephalomyelitis mice

Microglia and astrocytes produce key extracellular matrix regulators, MMP-2/9, and their specific inhibitors TIMP-1/2 (Kumnok et al., 2008). While MMPs degrade basal lamina proteins (compromising BBB/BSCB integrity) (Ying et al., 2020; Zhu et al., 2023), TIMPs maintain barrier function by inhibiting MMP activity (Su et al., 2020). TIMP-1 inhibits MMP-9 and TIMP-2 targets MMP-2 (Avolio et al., 2005). The MMP/TIMP imbalance drives EAE progression (Pyka-Fosciak et al., 2020).

**Figure 13:**
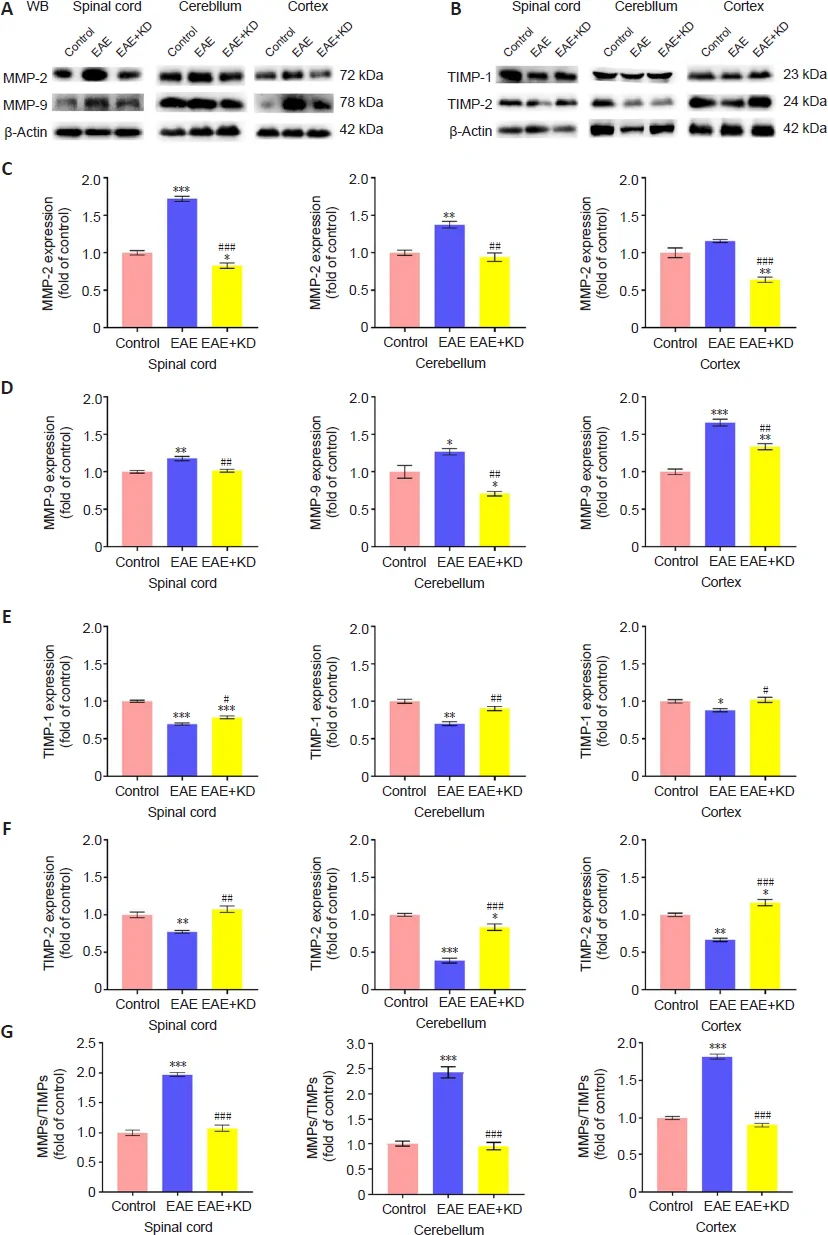
Effects of KD on the expression of MMPs (MMP-2 and MMP-9) and TIMPs (TIMP-1 and TIMP-2) in the spinal cord, cerebellum and cortex of mice. (A) Representative western blot images of MMP-2 and MMP-9 expression. (B) Representative western blot images of TIMP1 and TIMP2 expression. β-actin was used as the internal control for normalization. (C, D) Quantitative analysis of the expression of MMPs, including MMP-2 (C) and MMP-9 (D), in the spinal cord, cerebellum and cortex. (E, F) Quantitative analysis of the expression of TIMPs, including TIMP-1 (E) and TIMP-2 (F), in the spinal cord, cerebellum, and cortex. (G) Quantitative analysis of the ratio of MMPs/TIMPs. Data are presented as the mean ± SEM (*n* = 4). *P < 0.05, **P < 0.01, ***P < 0.001, *vs.* control group; #*P* < 0.05, ##*P* < 0.01, ###*P* < 0.001, *vs.* EAE group (one-way analysis of variance with Tukey’s *post hoc* test). EAE: Experimental autoimmune encephalomyelitis; KD: ketogenic diet; MMP: matrix metalloproteinase; TIMP: tissue inhibitor of metalloproteinase.

Western blot analysis revealed distinct patterns of MMP/TIMP regulation across CNS regions (**Figure 13**). Compared with controls, EAE mice exhibited elevated MMP-2 (spinal cord, *P* < 0.001; cerebellum, *P* < 0.01) and MMP-9 (spinal cord, *P* < 0.01; cerebellum, *P* < 0.05; cortex, *P* < 0.001) expression; these levels were significantly attenuated by KD administration (MMP-2 [spinal cord, *P* < 0.001; cerebellum, *P* < 0.01; cortex, *P* < 0.001] and MMP-9 [spinal cord, *P* < 0.01; cerebellum, *P* < 0.01; cortex, *P* < 0.01]; **Figure 13A**, **C**, and **D**). Moreover, KD administration enhanced TIMP-1 (spinal cord, *P* < 0.05; cerebellum, *P* < 0.01; cortex, *P* < 0.05) and TIMP-2 (spinal cord, *P* < 0.01; cerebellum, *P* < 0.001; cortex, *P* < 0.001) levels relative to levels in EAE mice (**Figure 13B**, **E**, and **F**). This coordinated modulation resulted in significant restoration of the MMP/TIMP ratio (spinal cord, *P* < 0.001; cerebellum, *P* < 0.001; cortex, *P* < 0.001, **Figure 13G**), indicating the capacity of the KD to rebalance this critical protease-antiprotease system in neuroinflammation.

### Ketogenic diet administration decreases the expression of leukocyte/monocyte-associated chemokines and chemokine receptor in the spinal cord, cerebellum, and cortex of experimental autoimmune encephalomyelitis mice

**Figure 14:**
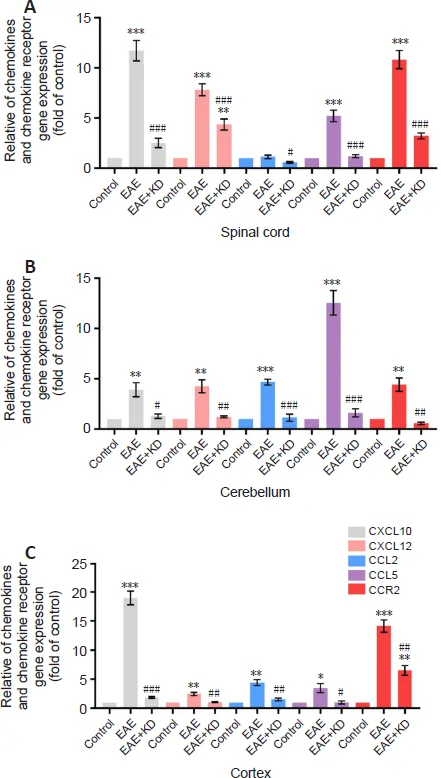
Effects of KD on leukocyte/monocyte-associated chemokines and chemokine receptors in the spinal cord, cerebellum, and cortex of mice. (A, B) Quantitative reverse transcription-polymerase chain reaction analysis of CXCL10, CXCL12, CCL2, CCL5 and CCR2 mRNA transcripts in the spinal cord (A), cerebellum (B), and cortex (C). Data are presented as the mean ± SEM (*n* = 4). *P < 0.05, **P < 0.01, ***P < 0.001, *vs*. control group; #*P* < 0.05, ##*P* < 0.01, ###*P* < 0.001, *vs*. EAE group (one-way analysis of variance with Tukey’s *post hoc* test). EAE: Experimental autoimmune encephalomyelitis; KD: ketogenic diet.

Chemokine signaling facilitates peripheral immune cell infiltration across the compromised BBB/BSCB in MS/EAE, exacerbating disease through pro-inflammatory cytokine release. We examined the effects of KD on critical chemotactic axes. The C-C motif chemokine receptor 2 (CCR2)–C-C motif chemokine ligand 2 (CCL2) chemokine axis plays a pivotal role in mediating inflammatory leukocyte infiltration into the CNS during MS/EAE pathogenesis (Errede et al., 2022). C-C motif chemokine ligand 5 (CCL5) contributes to monocyte recruitment and activation (Monaghan et al., 2019), while glial-derived C-X-C motif chemokine ligand 10 (CXCL10) facilitates leukocyte migration (Li et al., 2020a). CXCL12 overexpression at inflammatory sites promotes monocyte transmigration across the BBB (Zilkha-Falb et al., 2016). RT-PCR analysis demonstrated that while EAE mice exhibited upregulated mRNA expression of the key chemotactic molecules CXCL10 (spinal cord, *P* < 0.001; cerebellum, *P* < 0.01; cortex, *P* < 0.001), CXCL12 (spinal cord, *P* < 0.001; cerebellum, *P* < 0.01; cortex, *P* < 0.01), CCL2 (cerebellum, *P* < 0.001; cortex, *P* < 0.01), CCL5 (spinal cord, *P* < 0.001; cerebellum, *P* < 0.001; cortex, *P* < 0.05), and receptor CCR2 (spinal cord, P < 0.001; cerebellum, *P* < 0.01; cortex, *P* < 0.001) in the CNS compared with controls, KD administration effectively suppressed their expression (CXCL10 [spinal cord, *P* < 0.001; cerebellum, *P* < 0.05; cortex, *P* < 0.001], CXCL12 [spinal cord, *P* < 0.001; cerebellum, *P* < 0.01; cortex, *P* < 0.01]; CCL2 [spinal cord, *P* < 0.05; cerebellum, *P* < 0.001; cortex, *P* < 0.01], CCL5 [spinal cord, *P* < 0.001; cerebellum, *P* < 0.001; cortex, *P* < 0.05]; CCR2 [spinal cord, *P* < 0.001; cerebellum, *P* < 0.01; cortex, *P* < 0.01]; **Figure 14A–C**). These results suggested that the KD inhibits the expression levels of chemokines/chemokine receptor in the spinal cord, cerebellum and cortex of EAE mice, thereby impairing the molecular pathways critical for leukocyte migration.

## Discussion

BBB and BSCB are selective physical barriers with protective roles in maintaining brain homeostasis (Jin et al., 2021). Research has recently shown a relation of BBB/BSCB alterations with MS pathology (Zhang et al., 2021). A deep insight into the complexity and diversity of BBB/BSCB modulators is critical for understanding the pathogenesis of MS. BBB/BSCB integrity is regulated by various factors, such as glial-BBB/BSCB interactions and inflammatory cytokines/chemokines. In this study, our findings demonstrate that the KD exerts its therapeutic effects through (1) inhibiting demyelination, (2) maintaining integrity of the BBB/BSCB by promoting the expression of TJ proteins (ZO-1, Occludin, Claudin-5, and Claudin-1) and AJ proteins (VE-cadherin and β-catenin), (3) inhibiting M1-type-microglia/A1-type-astrocyte polarization and promoting M2-type-microglia/A2-type-astrocyte polarization, (4) modulating MMP/TIMP ratio, and (5) downregulating the expression of chemokines/chemokine receptors, which guide immune/inflammatory cell migration. Our study demonstrated that KD administration significantly suppressed MAPK signaling pathway activity, which is mechanistically linked to reduced expression of BBB/BSCB-associated proteins. KD administration attenuated the HDAC3/STAT3/NF-κB/NLRP3 pro-inflammatory axis while activating the PI3K/AKT neuroprotective pathway, collectively orchestrating astrocytic phenotypic switching from A1 to A2 in EAE mice.

Growing evidence supports the neuroprotective potential of KD in neurodegenerative diseases, including MS (Bahr et al., 2020; Gough et al., 2021; Hersant and Grossberg, 2022). Research has elucidated several potential mechanisms underlying these benefits, including: (1) attenuation of neuroinflammation, (2) promotion of remyelination, (3) optimization of neuronal energy metabolism, and (4) modulation of microglial activation states. Elevated blood ketone levels have also been implicated in BBB restoration through upregulation of monocarboxylate transporters and glucose transporter expression, thereby reinforcing barrier integrity (Jensen et al., 2020; NICE Guideline Committee, 2022). Supporting this, Versele et al. (2020) showed that KD and its metabolic mediators, ketone bodies, act at the BBB level to facilitate clearance of cerebral amyloid plaques. Collectively, these findings substantiate a role for KD in preserving BBB/BSCB and protecting the nervous system.

The disruption of the BBB/BSCB is an early pathological event in MS that precedes demyelination (Nishihara et al., 2022). TJs and AJs such as ZO-1, Claudin-1, Occludin, VE-cadherin, and β-catenin are essential for barrier function, and their loss correlates with disease severity (Huang et al., 2022). Inhibition of MAPK pathways (ERK1/2, JNK, and p38) increases the expression of TJ proteins, such as Claudin-5, ZO-1, and Occludin (Li et al., 2013; Xu et al., 2013). Our findings show that KD administration enhanced BBB and BSCB integrity in EAE mice by increasing ZO-1 and Occludin levels partly by inhibition of MAPK pathways (ERK1/2, JNK, and p38).

Astrocytes, the predominant glial population in the CNS, serve crucial functions in neural development and homeostasis and are emerging therapeutic targets (Yang et al., 2023). In MS, reactive astrocytes polarize into two functionally distinct phenotypes: the neurotoxic A1 type, which releases pro-inflammatory cytokines (such as TNF-α and IL-6) and cytotoxic factors that disrupt myelin, neurons, and vascular barriers; and the neuroprotective A2 type, which helps resolve inflammation and maintain barrier integrity (Ding et al., 2021; Wu et al., 2021; Zhang et al., 2022). Therapeutic modulation of astrocyte activation toward the A2 phenotype may preserve BBB/BSCB integrity and attenuate EAE progression. The activation of A1 astrocytes is primarily mediated by the key signaling molecules NLRP3, NF-κB, and STAT3. These pathways collectively drive the release of pro-inflammatory cytokines and MMPs not only from A1 astrocytes but also from M1 microglia, sustaining CNS neuroinflammation (Deng et al., 2021; Fan and Huo, 2021; Zhang et al., 2022; Sun et al., 2023). Importantly, this activation state can be therapeutically modulated, as demonstrated by MFG-E8, which can transform neurotoxic A1 astrocytes into protective A2 astrocytes by suppressing the NF-κB and PI3K/Akt pathways. Nlrp3^–/–^ microglial conditioned media blocked the A1 astrocytic gene response to LPS/ATP (Li et al., 2022). NLRP3-deficient mice exhibited significantly attenuated astrogliosis and microglial activation in a cuprizone-induced demyelination model compared with wild-type controls (Freeman et al., 2017). Pang et al. (2023) reported that M2 microglia promotes astrocyte polarization to type A2 via the PI3K/AKT signaling pathway. Here, our results establish that the KD preserves BBB/BSCB integrity in EAE mice by modulating astrocyte polarization from A1 to A2 through inhibition of STAT3/NF-кB/NLRP3 signaling pathways and activation of PI3K/AKT signaling pathway, thereby extending our previous discovery of KD-mediated NLRP3 suppression and aligning with our recent work (Zhang et al., 2025). Additionally, GFAP/NLRP3 co-localization analysis demonstrated attenuated NLRP3 expression in astrocytes in the EAE + KD group, confirming NLRP3’s participation in astrocyte activation and its inhibitory effect on A1 polarization. Neuroinflammation in the CNS involves complex glial crosstalk that critically regulates immune responses and neural repair processes in MS and EAE (Jha et al., 2019; Greenhalgh et al., 2020). Li et al. (2022) demonstrated that microglial NF-κB activation can trigger NLRP3 inflammasome assembly and induce A1 astrocyte formation (Li et al., 2022). Moreover, microglia-mediated downregulation of the CXCR7/PI3K/Akt pathway was shown to drive A1 astrocyte polarization in the spinal cord, a mechanism implicated in chronic post-surgical pain development (Li et al., 2020b). Our previous study showed that M2 type microglia plays a protective role in BBB integrity through downregulation of HDAC3/NF-κB/NLRP3 signaling pathways (Zhang et al., 2023). In line with this, our current results indicate that KD administration shifted microglia toward an M2 phenotype, lowering pro-inflammatory cytokines (TNF-α and IL-6) and elevating anti-inflammatory cytokines (IL-4 and TGF-β) in the CNS, suggesting that astrocyte repolarization is partly microglia-dependent.

MMPs contribute to BBB/BSCB breakdown through proteolytic degradation of key extracellular matrix components, including fibronectins, laminins, and TJ proteins (Zapata-Acevedo et al., 2022). In response to inflammatory signals, local MMPs degrade the neurorestrictive barrier, thereby enabling leukocyte infiltration into the CNS parenchyma (Glaser et al., 2022). In the CNS, MMPs are synthesized and released by neurons, astrocytes and microglia (Zhang et al., 2022), with MMP-2 and MMP-9 playing particularly critical roles in immune cell recruitment and barrier breakdown, their absence has been shown to inhibit EAE (Ying et al., 2020). Treatment with DL-3-n-butylphthalide inhibited the activation of MMP-2 and MMP-9, leading to upregulated expression of TJs Claudin-5 and ZO-1 and decreased BBB leakage in bilateral common carotid artery stenosis mice (Han et al., 2019). Similarly, selective HDAC3 inhibition with RGFP966 alleviates cerebral edema and BBB leakage in a cuprizone-induced demyelination model by downregulating MMP-9 and upregulating ZO-1, Occludin, and VE-cadherin (Zhao et al., 2019; Lu et al., 2023). TIMPs are crucial regulators of MMP activity; TIMP-1 preferentially inhibits MMP-9, while TIMP-2 more effectively targets MMP-2 (Zakłos-Szyda and Budryn, 2020). Genetic evidence from TIMP-1-deficient mice demonstrated that MMP-9 dysregulation leads to BBB disruption and neuronal apoptosis during ischemic injury (Fujimoto et al., 2008), highlighting the critical importance of maintaining MMP/TIMP homeostasis for BBB/BSCB integrity. Our data reveal that the KD effectively rebalances this system in EAE mice, significantly reducing MMP-2/9 levels while elevating TIMP-1/2 expression across CNS regions. These findings suggest that KD-mediated protection of BBB/BSCB integrity may occur through normalization of the MMP/TIMP equilibrium.

Neuroinflammatory processes are central to MS pathogenesis (Sun et al., 2023). Pro-inflammatory cytokines (e.g., TNF-α, IL-1β, and IL-6) mediate BBB/BSCB disruption in EAE models (Wang et al., 2020). In this study, we observed a marked reduction in TNF-α, IL-1β, and IL-6 expression in EAE + KD mice. Several reports have indicated that chemotaxis molecules (e.g., CCL5, CCL2, and CCR2) lead to increased permeability of BBB/BSCB and leukocyte/monocyte migration into the CNS, which aggravate MS and EAE symptoms (Huang et al., 2022; Lin et al., 2023). Notably, CCL2 and CCL5 facilitate leukocyte adhesion to cerebral microvasculature, a critical step in EAE development (dos Santos et al., 2005; Zaidan et al., 2022). CXCL10 enhances the transmigration of monocytes across the BBB/BSCB *in vitro* and *in vivo* (Li et al., 2020; Constant et al., 2023), while CXCL12 mediates lymphocyte transmigration and contributes to neuronal inflammation in MS (Hansmann et al., 2012). Our findings demonstrate that KD administration significantly downregulates key chemotactic mediators (CXCL10, CXCL12, CCL2, CCL5, and CCR2) in EAE mice. This suppression of chemokine/chemokine receptor expression suggests that the KD may attenuate neuroinflammation by impeding leukocyte/monocyte trafficking into the CNS.

The current study demonstrates that the KD provides neuroprotection in EAE mice by preserving BBB/BSCB integrity through enhanced the expression of TJ and AJ, promoting A1-to-A2 astrocyte transformation, rebalancing MMP/TIMP levels, and modulating the HDAC3/STAT3/NF-κB/NLRP3 and PI3K/AKT pathways, suggesting its therapeutic potential for MS. However, several limitations warrant consideration. For instance, the relatively small sample size per group. Additionally, species differences in ketone metabolism may limit clinical translation and the individual contributions of the modulated signaling pathways have not been isolated. Future studies should therefore integrate mechanistic metabolomics, pathway-specific genetic approaches, and human trials with neuroimaging validation to fully elucidate KD’s therapeutic mechanism and clinical applicability.

## Additional files:

***Additional Table 1:*** *Composition of the SD and KD used in this study.*

***Additional Table 2:*** *Antibodies used for western blot analysis.*

***Additional Table 3:*** *Primer sequences for quantitative reverse transcription-polymerase chain reaction.*

***Additional Table 4:*** *Levels of* β*-HB in the spinal cord, cerebellum, and cortex.*

***Additional Figure 1:*** *Immunohistochemical staining for tight junction markers (claudin-5 and occludin) in the spinal cord, cerebellum, and cortex.*

Additional Figure 1 Immunohistochemical staining for tight junction markers (claudin-5 and occludin) in the spinal cord, cerebellum, and cortex.
(A, C, E) Representative immunohistochemical staining images of claudin5 in the spinal cord (A), cerebellum (C), and cortex (E). (B, D, F) Quantitative analysis of the claudin-5-positive area in the spinal cord (B), cerebellum (D), and cortex (F). (G, I, K) Representative immunohistochemical staining images of occludin in the spinal cord (G), cerebellum (I), and cortex (K). (H, J, L) Quantitative analysis of the occludin-positive area in the spinal cord (H), cerebellum (J), and cortex (L). Data are presented as the mean ± SEM (*n* = 4). ^*^P < 0.05, ^**^P < 0.01, ^***^P < 0.001, *vs.* control; ^##^*P* < 0.01, ^###^*P* < 0.001, *vs*. EAE.

***Additional Figure 2:*** *Immunofluorescent double staining for GFAP*^*+*^*/PI3K*^*+*^ *cells and GFAP*^*+*^*/AKT*^*+*^ *cells in mice.*

Additional Figure 2 Immunofluorescent double staining for GFAP^+^/PI3K^+^ cells and GFAP^+^/AKT^+^ cells in mice.
(A, C, E) Representative images of double immunofluorescence staining of GFAP^+^/PI3K^+^ cells in the spinal cord (A), cerebellum (C), and cortex (E). (B, D, F) The quantification of GFAP^+^/PI3K^+^ cells in the spinal cord (B), cerebellum (D), and cortex (F) of the three groups is presented as the relative mean gray value. (G, I, K) Representative images of double immunofluorescence staining of GFAP^+^/AKT^+^ cells in the spinal cord (G), cerebellum (I), and cortex (K). (H, J, L) The quantification of GFAP^+^/AKT^+^ cells in the spinal cord (H), cerebellum (J), and cortex (L) of the three groups is presented as the relative mean gray value. Data are presented as the mean ± SEM (*n* = 4). *P < 0.05, **P < 0.01, ***P < 0.001, *vs*. control group; #*P* < 0.05, ##*P* < 0.01, ###*P* < 0.001, *vs*. EAE group (one-way analysis of variance with Tukey’s post hoc test). DAPI: 4′,6-Diamidino-2-phenylindole; EAE: experimental autoimmune encephalomyelitis; GFAP: glial fibrillary acidic protein; KD: ketogenic diet.

## Data Availability

*All relevant data are within the paper and its Additional files.*
